# Mapping the transition state for a binding reaction between ancient intrinsically disordered proteins

**DOI:** 10.1074/jbc.RA120.015645

**Published:** 2020-10-16

**Authors:** Elin Karlsson, Cristina Paissoni, Amanda M. Erkelens, Zeinab A. Tehranizadeh, Frieda A. Sorgenfrei, Eva Andersson, Weihua Ye, Carlo Camilloni, Per Jemth

**Affiliations:** 1 Department of Medical Biochemistry and Microbiology, Uppsala University, Uppsala, Sweden; 2 Dipartimento di Bioscienze, Università degli Studi di Milano, Milano, Italy; 3 Department of Medicinal Chemistry, School of Pharmacy, Mashhad University of Medical Sciences, Mashhad, Iran

**Keywords:** intrinsically disordered proteins, phi value analysis, transition state, protein evolution, coupled binding and folding, protein folding, pre-steady-state kinetics, protein complex, IDP, protein binding

## Abstract

Intrinsically disordered protein domains often have multiple binding partners. It is plausible that the strength of pairing with specific partners evolves from an initial low affinity to a higher affinity. However, little is known about the molecular changes in the binding mechanism that would facilitate such a transition. We previously showed that the interaction between two intrinsically disordered domains, NCBD and CID, likely emerged in an ancestral deuterostome organism as a low-affinity interaction that subsequently evolved into a higher-affinity interaction before the radiation of modern vertebrate groups. Here we map native contacts in the transition states of the low-affinity ancestral and high-affinity human NCBD/CID interactions. We show that the coupled binding and folding mechanism is overall similar but with a higher degree of native hydrophobic contact formation in the transition state of the ancestral complex and more heterogeneous transient interactions, including electrostatic pairings, and an increased disorder for the human complex. Adaptation to new binding partners may be facilitated by this ability to exploit multiple alternative transient interactions while retaining the overall binding and folding pathway.

Intrinsically disordered proteins (IDPs) are abundant in the human proteome (1) and are frequently involved in mediating protein-protein interactions in the cell. The functional advantages of disordered proteins include exposure of linear motifs for association with other proteins, accessibility for post-translational modifications, formation of large binding interfaces, and the ability to interact specifically with multiple partners. These properties make IDPs suitable for regulatory functions in the cell, for example as hubs in interaction networks governing signal transduction pathways and transcriptional regulation (2).

The ability to mutate while maintaining certain sequence characteristics might allow IDPs to more efficiently explore sequence space, facilitating adaptation to new binding partners. Despite recent appreciation of the biological importance of IDPs and progress in understanding their mechanism of interaction with other proteins, the changes that take place at a molecular level when these proteins evolve to accommodate new binding partners remain elusive. The reason for this is the inherent difficulty in assessing effects of mutations that a protein has acquired during millions or billions of years. However, the rapidly increasing number of available protein sequences from extant species has enabled the development of ancestral sequence reconstruction as a tool for inferring the evolutionary history of proteins (3). In combination with biophysical characterization of the resurrected proteins, this method has been employed successfully to deduce details in the evolution of numerous proteins (4–10). Ancestral sequence reconstruction relies on an alignment of sequences from extant species and a maximum likelihood method that infers probabilities for amino acids at each position in the reconstructed ancestral protein from a common ancestor.

However, because they have fewer constraints for maintaining a folded structure, IDPs are often thought to experience faster amino acid substitution rates during evolution and an increased occurrence of amino acid deletion and insertion events as compared with folded proteins. In particular, deletions and insertions obstruct reliable sequence alignments of IDPs (11–13). Nevertheless, as pointed out before (14), IDPs do not constitute a homogenous group of proteins, and thus the degree of sequence conservation differs among different classes of IDPs. IDP regions that form binding interfaces in coupled binding and folding interactions are usually conserved because of sequence restraints for maintaining affinity and structure of the protein complex (15) and can therefore be subjected to ancestral sequence reconstruction.

The nuclear coactivator binding domain (NCBD) from CREB-binding protein (CBP) is engaged in multiple protein-protein interactions in the cell (16). NCBD is a molten globule-like protein (17), which forms three helices in the unbound state that rearranges upon binding to its different partners (18–20). These binding partners include, among others, the transcription factors and transcriptional coregulators p53, IRF3, and NCOA1, -2, and -3 (also called SRC, TIF2, and ACTR, respectively). The interaction between NCBD and the CREBBP-interacting domain (CID) from NCOA3 (ACTR) has been intensively studied with kinetic methods to elucidate details about the binding mechanism (21–24). These protein domains interact in a coupled binding and folding reaction in which CID forms two to three helices that wrap around NCBD (18). The binding reaction involves several steps, as evidenced from the multiple kinetic phases observed in stopped-flow spectroscopy and single molecule-FRET experiments (24–26). It is, however, not clear what structural changes all kinetic phases correspond to. In fact, equilibrium data agree well with *K_d_* calculated from the major kinetic binding phase (22) in overall agreement with a two-state binding mechanism.

Previous phylogenetic analyses revealed that whereas NCBD was present already in the last common ancestor of all bilaterian animals (deuterostomes and protostomes), CID likely emerged later as an interaction domain within the ancestral ACTR/TIF2/SRC protein in the deuterostome lineage (including chordates, echinoderms, and hemichordates) (Fig. 1) (4, 27). The evolution of the NCBD/CID interaction was previously examined using ancestral sequence reconstruction in combination with several biophysical methods to assess differences in affinity, structure, and dynamics between the extant and ancestral protein complex. After the emergence of CID in the ancestral ACTR/TIF2/SRC protein, NCBD increased its affinity for CID while maintaining the affinity for some of its other binding partners (4). Although the overall structure of the ancestral NCBD/CID complex, which we denote as “Cambrian-like,” is similar to the modern high-affinity human one, there are several differences on the molecular level (Fig. 1) (28).

**Figure 1.**
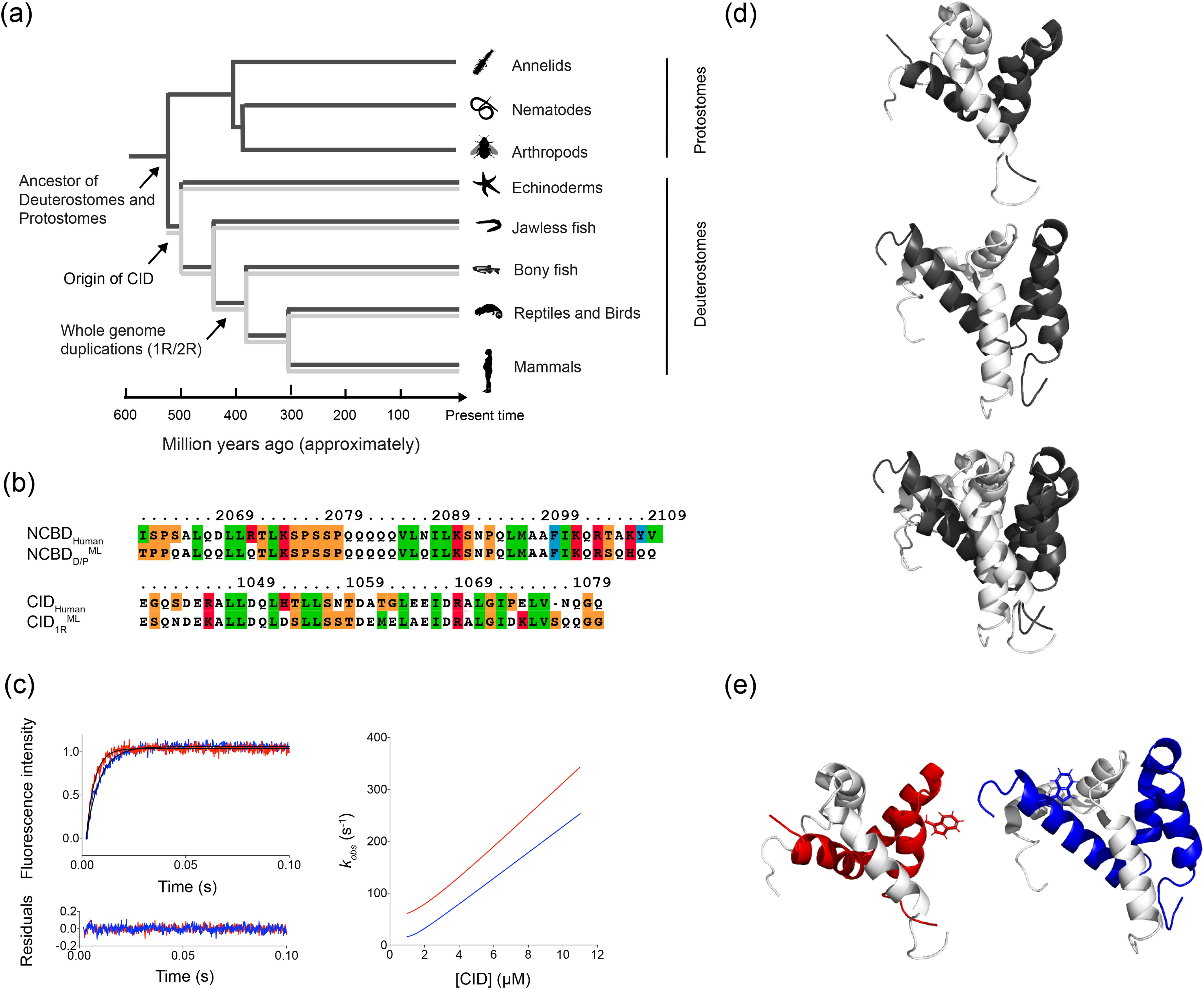
**Extant and ancestral NCBD and CID variants.** *a*, a schematic phylogenetic tree showing the evolutionary relationship between extant species and nodes corresponding to ancestral species for which ancestral NCBD (*dark gray*) and CID (*light gray*) variants were reconstructed. The animal pictures were obtained from PhyloPic, RRID:SCR_019139. *b*, the sequences of the reconstructed ancestral and extant human NCBD (*top*) and CID (*bottom*) that were used in the study. Human NCBD is from CBP and human CID is from NCOA3/ACTR. The color denotes residue type. *c*, examples of typical stopped-flow kinetic traces for the Cambrian-like complex (*red*) and human complex (*blue*; *left panel*). The concentrations used in this example were 1 µm NCBD and 6 µm CID for both experiments. The kinetic traces were fitted to a single exponential function (shown as a *solid black line*) and the residuals are displayed below the curve. *Right panel*: the dependence of the observed rate constant (*k*_obs_) on CID concentration for the Cambrian-like complex (*red*) and the human complex (*blue*), calculated using the rate constants obtained in global fitting (Table 3). *d*, solution structures of the Cambrian-like complex (*top*; PDB entry 6ES5), the human complex (*middle*; PDB entry 1KBH), and an alignment of the two complexes (*bottom*) with NCBD in *dark gray* and CID in *light gray*. *e*, structures of the Cambrian-like complex (*left*; NCBD in *red* and CID in *light gray*) and the extant human complex (*right*; NCBD in *blue* and CID in *light gray*) showing the position of the engineered Trp residues as stick model.

Here, we address the molecular details of the evolutionary optimization of the binding energy landscape between NCBD and CID. More specifically, we have investigated evolutionary changes of the binding transition state (TS) by subjecting the Cambrian-like NCBD/CID complex to site-directed mutagenesis and kinetic measurements using fluorescence-monitored stopped-flow spectroscopy. The Cambrian-like complex consisted of the maximum likelihood estimate (ML) NCBD variant from the time of the divergence between deuterostomes and protostomes, NCBD_D/P_^ML^, and the CID variant from the time of the first whole genome duplication, CID_1R_^ML^. The experimental data were used to estimate the degree of native intermolecular tertiary contacts and helical content of CID in the transition state, expressed as ϕ-values. The ϕ-value, which commonly ranges from 0 to 1, reports on formation of native contacts in the transition state and was originally developed for folding studies (29). However, it has also been used to gain detailed information about the transition state structures for several IDP binding reactions (30). Furthermore, to obtain an atomic-level detail of the binding and folding process, the ϕ-values were employed for restrained molecular dynamics (MD) simulations. Our results suggest that although the overall binding mechanism and transition state are conserved, some modulation of helical content in the transition state and, most notably, an increased heterogeneity of transient intermolecular interactions are observed in the high-affinity modern human complexes as compared with the low-affinity Cambrian-like complex.

## Results

### Experimental strategy

**Figure 2.**
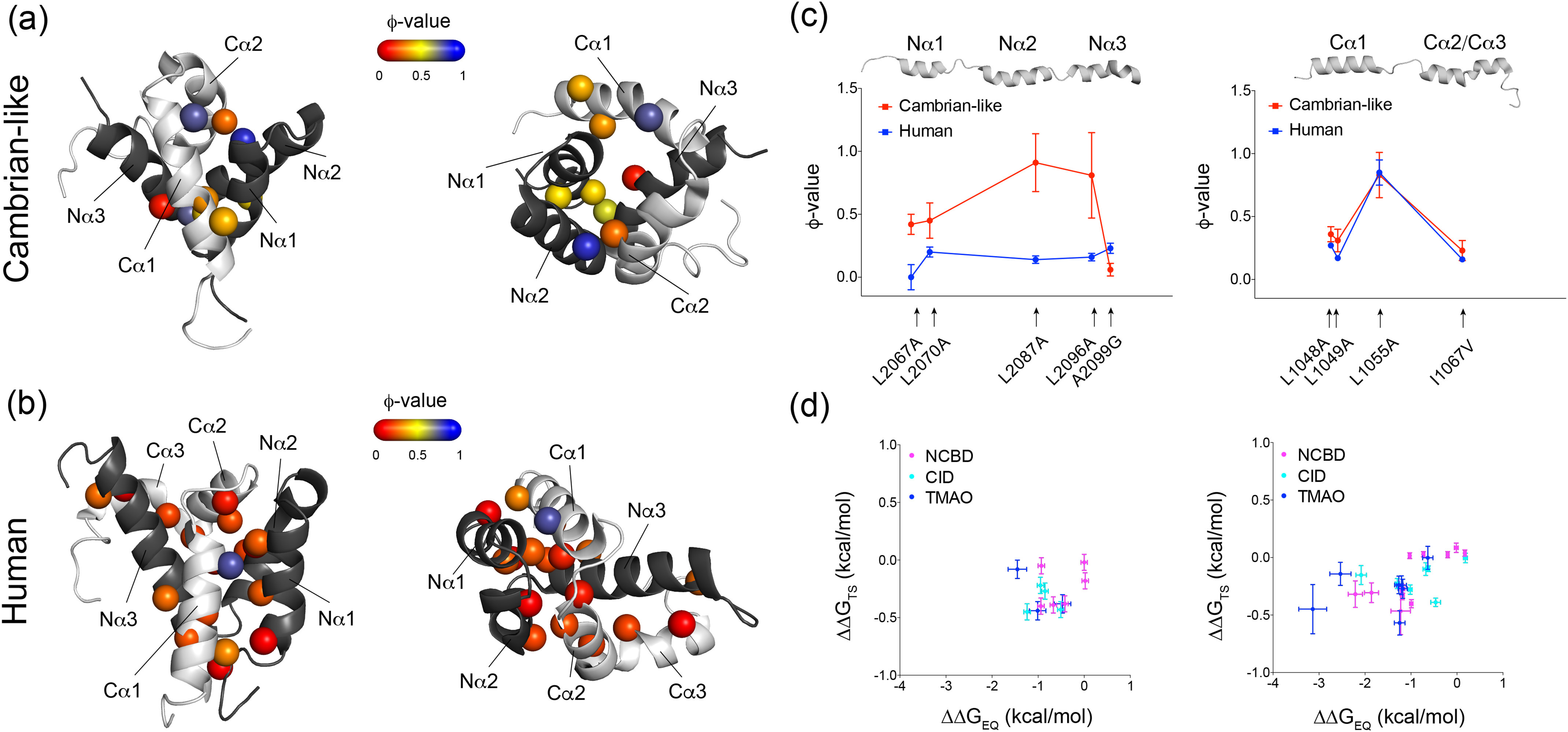
**ϕ-values mapped onto the structures of the Cambrian-like and human complexes.** *a*, ϕ-values for conservative deletion mutations (mostly Leu → Ala mutations) in the binding interface of the Cambrian-like complex. NCBD in *dark gray* and CID in *light gray*. The two structures represent the same complex from different angles. Most ϕ-values fall within the intermediate to high ϕ-value category (0.3–0.9; Dataset S1*A*). *b*, the previously published ϕ-values for conservative deletion mutations in the binding interface of the human NCBD/CID complex. Most ϕ-values are in the low region (<0.3). *c*, a site-to-site comparison between ϕ-values at corresponding positions in the Cambrian-like (*red*) and human (*blue*) complexes. The *error bars* denote propagated standard errors. *d*, Brønsted plots for the Cambrian-like (*left*; Dataset S1*B*) and human (*right*; Dataset S1*C*) NCBD/CID interaction. Data for human NCBD/CID were obtained from previous studies. The *error bars* are propagated standard errors. All structures were created using PyMOL.

We have characterized the transition state of binding for the low-affinity Cambrian-like NCBD/CID complex in terms of ϕ-values and compared it to that of the high-affinity modern human complex. A ϕ-value can be employed as a region-specific probe for native contact formation in the transition state of a binding reaction. A ϕ-value of 0 means that the entire effect on *K_d_* from the mutation stems from a change in *k*_off_, indicating that the interactions with the mutated residue mainly forms after the transition state barrier of binding. On the other hand, a ϕ-value of 1 is obtained when the effect of mutation on *k*_on_ and *K_d_* is equally large, which suggests that the mutated residue is making fully formed native contacts at the top of the transition state barrier. To characterize the transition state for binding, we performed extensive site-directed mutagenesis in the Cambrian-like complex and subjected each mutant to stopped-flow kinetic experiments to obtain binding rate constants (*i.e. k*_on_ and *k*_off_) (Fig. 1*c*). These rate constants were used to compute ϕ-values for each mutated position in the complex (Fig. 2*a*). To provide more structural details about the evolution of the NCBD/CID interaction, we also determined the TS ensemble of the Cambrian-like complex via ϕ-value–restrained MD simulations, following the same procedure previously used to obtain the TS ensemble of the human complex (23).

In the present study, we used NCBD_D/P_^T2073W^ as a pseudo-WT (Fig. 1*e*; denoted NCBD_D/P_^pWT^) to obtain a sufficient signal change in the stopped-flow fluorescence measurements. Although Trp pseudo-WTs have been shown to be reliable in protein-folding studies (31), it is less clear how much an engineered Trp would affect an IDP complex in a coupled binding and folding reaction. Therefore, we initially tested five different Trp mutants of NCBD_D/P_^ML^ to select the one with properties most similar to NCBD_D/P_^ML^ (Fig. S1). To check how robust our ϕ-values were to the position of the Trp probe, we measured five ϕ-values with one of the other Trp variants, NCBD_D/P_^L2067W^. Despite a 2-fold lower *k*_on_ as compared with NCBD_D/P_^pWT^ (*i.e.* NCBD_D/P_^T2073W^), all five ϕ-values were very similar, including the negative ϕ-value obtained for D1068A (Table 1), suggesting that our experimental ϕ-values are robust and not dependent on the optical probe.

**Table 1:** **Rate constants of binding of NCBD_D/P_^L2067W^ to different CID variants determined in stopped-flow experiments** The experiments were performed once for each mutant, and an error of 10% was applied on all *k_on_* values. The errors in *k_off_* are standard errors from global fitting of the kinetic data to a two-state model (Dataset S1*A*). All experiments were performed in 20 mm sodium phosphate, pH 7.4, and 150 mm NaCl at 4 °C. In general, the resulting ϕ-values are similar to the ones obtained using the NCBD_D/P_^pWT^ variant with Trp-2073 (shown in parentheses).

NCBD_D/P_^pWT^ variant	CID_1R_ variant	*k*_on_ (µm^−1^ s^−1^)	*k*_off_ (s^−1^)	Type of mutation	*K_d_* (µm)	ϕ-value
L2067W	ML	17.9 ± 1.8	39.2 ± 0.2	-	2.2 ± 0.2	-
L2067W	A1047G	12.9 ± 1.3	64.7 ± 0.6	Helix-modulating Cα1	5.0 ± 0.5	0.40 ± 0.18 (0.21 ± 0.13)
L2067W	L1049A	9.6 ± 0.96	52.9 ± 0.2	Native interactions with Leu-2071, Leu-2074, and Phe-2100	5.5 ± 0.6	0.68 ± 0.18 (0.31 ± 0.09)
L2067W	D1053A	29.3 ± 2.9	25.7 ± 0.1	Electrostatic interaction with Lys-2075 and Arg-2104	0.88 ± 0.09	0.54 ± 0.18 (0.81 ± 0.32)
L2067W	I1067V	10.7 ± 1.1	132 ± 1	Native interactions with Leu-2074, Val-2086, Leu-2087, Leu-2090, and Phe-2099	12 ± 1	0.30 ± 0.09 (0.23 ± 0.08)
L2067W	A1065G	14.4 ± 1.4	125 ± 1	Helix-modulating Cα2	8.7 ± 0.9	0.16 ± 0.10 (0.05 ± 0.11)
L2067W	D1068A	27.0 ± 2.7	178 ± 2	Electrostatic interaction with Arg-2104	6.6 ± 0.7	−0.37 ± 0.14 (−0.43 ± 0.23)

We constructed two sets of mutants in this study to characterize the transition state for binding: one that targeted native contacts in the binding interface and a second that targeted native helices in CID. The first set consisted of 13 NCBD_D/P_^pWT^ variants and 10 CID_1R_^ML^ variants mainly corresponding to previously mutated residues in the human complex (21, 23). The positions spanned the entire binding interface of NCBD and CID to ensure that all regions of the protein domains were probed (Table 2). The majority of the mutations targeted interactions between hydrophobic residues in the binding interface; however, a few mutations also probed interactions between charged residues. The second set of mutants consisted of Ala → Gly substitutions in helices 1 and 2 of CID_1R_^ML^ and helices 2 and 3 of CID_Human_, complementing a previous dataset for helix-modulating mutations in helix 1 of CID_Human_ (32). The secondary structure content of all mutants was assessed with far-UV CD. All CID variants exhibited far-UV CD spectra typical for highly disordered proteins (Fig. S2, *A* and *B*). For NCBD, the far-UV CD measurements showed that NCBD_D/P_^L2070A^, NCBD_D/P_^L2090A^, NCBD_D/P_^L2096A^, and NCBD_D/P_^A2099G^ displayed substantially less α-helical structure than NCBD_D/P_^pWT^, as judged from the lower magnitude of the CD signal at 222 nm (Fig. S2*C*). Addition of 0.7 m trimethylamine *N*-oxide (TMAO) to the experimental buffer for these variants resulted in an increase in helical content such that the far-UV CD spectra of NCBD_D/P_^L2070A^ and NCBD_D/P_^L2096A^ were qualitatively similar to NCBD_D/P_^pWT^ (Fig. S2*D*). For NCBD_D/P_^L2096A^ and NCBD_D/P_^A2099G^, stopped-flow kinetic experiments were carried out both in regular buffer and in buffer supplemented with 0.7 m TMAO. The resulting ϕ-values were very similar for these variants at both conditions, demonstrating both the accuracy and precision of ϕ-values and how robust they are to different experimental conditions (Fig. S3). The complex of the NCBD_D/P_^L2070A^ variant was too unstable in buffer, and data were only recorded in presence of 0.7 m TMAO. Several mutants failed to generate kinetic data because of elevated *k*_obs_ values, which were too high to be reliably recorded with the stopped-flow technique. This was the case for NCBD_D/P_^L2074A^, NCBD_D/P_^L2086A^, NCBD_D/P_^L2090A^, NCBD_D/P_^I2101A^, CID_1R_^L1056A^, CID_1R_^L1064A^, CID_1R_^I1067A^, and CID_1R_^L1071A^.

**Figure 3.**
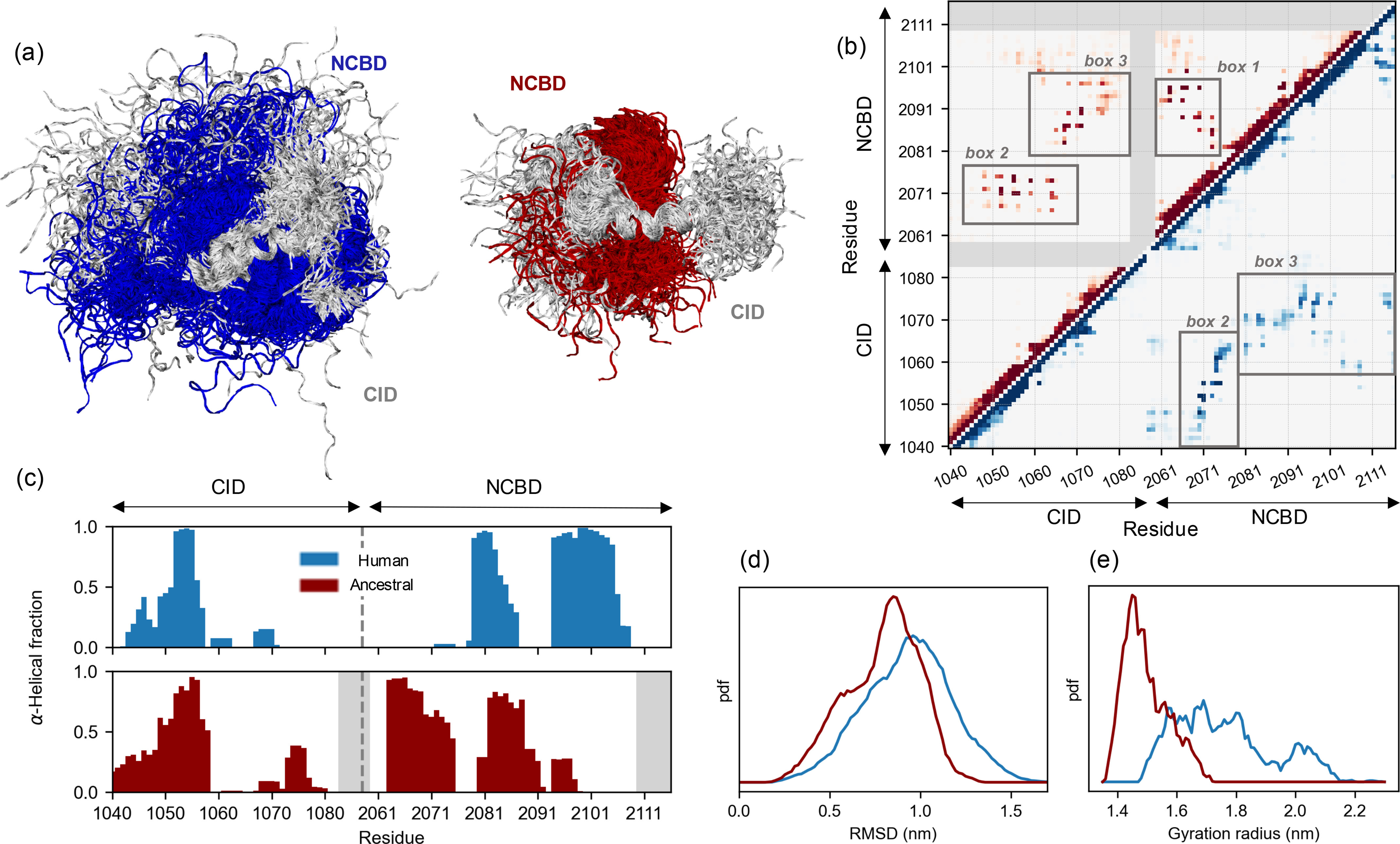
**Comparison of the transition state of human and ancestral Cambrian-like complexes.** *a*, MD-determined structural ensembles of the human (NCBD in *blue* and CID in *light gray*) and ancestral (NCBD in *red* and CID in *light gray*) TS. Both ensembles are aligned on CID helix Cα1. *b*, map representing the contact probability between each pair of residues in the human (*lower right*, *blue*) and in the ancestral (*upper left*, *red*) TS ensembles. Probability goes from 0 (*white*) to 1 (*dark blue*/*red*); regions involving residues which are not present in the ancestral complex are shaded with *gray*. *c*, per-residue α-helical content of the human and ancestral TS. *d* and *e*, probability distribution, in arbitrary units, of the root mean square deviation and of the gyration radius for the human (*blue*) and ancestral (*red*) TS ensembles.

**Table 2:** **Rate constants of binding for ancestral NCBD and CID variants determined in stopped-flow experiments** The mutants were either helix-modulating mutants of ancestral CID or deletion mutations in NCBD or CID chosen to probe for native inter- and intramolecular interactions in the ancestral complex (Dataset S1*A*). The rate constants were obtained from global fitting of the kinetic datasets to a simple two-state model by numerical integration. The NCBD_D/P_^pWT^/CID_1R_^ML^ complex was measured five times (biological replicates, *i.e* performed with different protein batches), and the error in *k_on_* is the standard deviation for these replicates. The NCBD and CID mutants were measured once, and the 10% error determined for the WT by replicate experiments was applied to these variants. The error in *k_off_* is the standard error from global fitting, except for the variants for which displacement experiments were performed. All experiments were performed in 20 mm sodium phosphate, pH 7.4, and 150 mm NaCl at 4 °C.

NCBD_D/P_ variant	CID_1R_ variant	*k*_on_ (µm^−1^ s^−1^)	*k*_off_ (s^−1^)	Type of mutation	*K_d_* (µm)	ϕ-value
pWT	ML	30.4 ± 2.5	24.7 ± 0.3*^a^*	-	0.81 ± 0.07	-
pWT	A1047G	24.8 ± 2.5	53.8 ± 0.8	Helix-modulating Cα1	2.17 ± 0.22	0.21 ± 0.13
pWT	D1050A	31.7 ± 3.2	21.3 ± 0.2*^a^*	Helix-modulating Cα1	0.67 ± 0.07	-
pWT	D1050G	18.7 ± 1.9	72 ± 0.9	Helix-modulating Cα1	3.85 ± 0.39	0.30 ± 0.08
pWT	S1054A	39.0 ± 3.9	14.9 ± 0.1*^a^*	Helix-modulating Cα1	0.38 ± 0.04	0.33 ± 0.18
pWT	S1054G	23.2 ± 2.3	74 ± 1	Helix-modulating Cα1	3.19 ± 0.32	0.24 ± 0.07
pWT	M1062A	29.8 ± 3.0	16.1 ± 0.3*^a^*	Helix-modulating Cα2	0.54 ± 0.05	−0.05 ± 0.32
pWT	M1062G	26.3 ± 2.6	21.2 ± 0.2*^a^*	Helix-modulating Cα2	0.81 ± 0.08	0.31 ± 0.37
pWT	A1065G	28.5 ± 2.9	77 ± 1	Helix-modulating Cα2	2.70 ± 0.27	0.05 ± 0.11
pWT	R1069A	26.0 ± 0.26	66.7 ± 0.6	Helix-modulating Cα2	2.57 ± 0.26	0.14 ± 0.11
pWT	R1069G	23.0 ± 2.3	209 ± 4	Helix-modulating Cα2	9.1 ± 0.9	0.10 ± 0.11
pWT	L1048A	13.5 ± 1.4	105 ± 1	Native interactions with Leu-2071	7.8 ± 0.8	0.36 ± 0.06
pWT	L1049A	18.7 ± 1.9	71.8 ± 0.6	Native interactions with Leu-2071, Leu-2074, and Phe-2100	3.84 ± 0.4	0.31 ± 0.09
pWT	D1053A	46.4 ± 4.6	22.3 ± 0.2*^a^*	Electrostatic interaction with Lys-2075 and Arg-2104	0.48 ± 0.05	0.81 ± 0.32
pWT	L1055A	14.0 ± 1.4	29.0 ± 0.4*^a^*	Native interactions with Leu-1064 and Leu-2074	2.07 ± 0.21	0.83 ± 0.18
pWT	L1056A	Too unstable complex	Too unstable complex	Native interactions with Leu-2074	-	-
pWT	L1064A	Too unstable complex	Too unstable complex	Native interactions with Leu-2074 and Phe-2100	-	-
pWT	I1067V	20.4 ± 2	93 ± 1	Native interactions with Leu-2074, Val-2086, Leu-2087, Leu-2090, and Phe-2099	4.56 ± 0.46	0.23 ± 0.08
pWT	I1067A	Too unstable complex	Too unstable complex	Native interactions with Leu-2074, Val-2086, Leu-2087, Leu-2090, and Phe-2100	-	-
pWT	D1068A	39.6 ± 4	59.5 ± 0.7	Electrostatic interaction with Arg-2104	1.50 ± 0.15	−0.43 ± 0.23
pWT	L1071A	Too unstable complex	Too unstable complex	Native interactions with Leu-2071, Leu-2074, Val-2086, Leu-2087, Leu-2090, Ala-2098, Ala-2099, and Ile-2101	-	-
L2067A	ML	14.8 ± 1.5	67 ± 1	Native interactions with Ile-2089, Leu-2090, and Leu-2096	4.5 ± 0.46	0.42 ± 0.08
L2071A	ML	22.1 ± 2.2	17.4 ± 0.1*^a^*	Native interactions with Leu2071, Ile2089 and Leu2093	0.79 ± 0.08	-
L2074A	ML	Too unstable complex	Too unstable complex	Native interactions with Leu-2071, Leu-2074, Leu-2078, Leu-2086, Ile-2089, Ala-2092, Leu-2093, and Ile-2095	-	-
K2075M	ML	14.5 ± 1.5	26.3 ± 0.4*^a^*	Electrostatic interaction with Asp-1053	1.81 ± 0.18	0.92 ± 0.22
K2075M	D1053A	18.0 ± 1.8	26.2 ± 0.2	-	1.46 ± 0.15	-
L2086A	ML	Too unstable complex	Too unstable complex	Native interactions with Ile-1067, Ala-1070, and Leu-1071	-	-
L2087A	ML	15.1 ± 1.5	26.4 ± 0.5*^a^*	Native interactions with Ala-1066, Ile-1067, Ala-1070, and Leu-1071	1.75 ± 0.2	0.91 ± 0.23
L2090A	ML	Too unstable complex	Too unstable complex	Native interactions with Ile-1067, Ala-1070, Leu-1071, and Ile-1073	-	-
L2096A	ML	14.9 ± 1.5	40.7 ± 0.5	Native interactions with Leu-2067 and Leu-2090	2.73 ± 0.28	0.59 ± 0.12
A2099G	ML	27.6 ± 2.8	122 ± 1	Native interactions with Leu-2067	4.42 ± 0.44	0.06 ± 0.08
I2101V	ML	29.3 ± 0.1	23.8 ± 0.3*^a^*	Native interactions with Leu-1071, Ile-1073, and Leu-1076	0.81 ± 0.01	
I2101A	ML	Too unstable complex	Too unstable complex	Native interactions with Leu-1071, Ile-1073, and Leu-1076	-	-
R2104M	ML	Too unstable complex	Too unstable complex	Native interactions with Asp-1068	-	-
R2104M	D1053A	Too unstable complex	Too unstable complex	-	-	-

*^a^* The dissociation rate constant was determined in a separate displacement experiment with the same experimental conditions as described above. The *k*_obs_ value at 20-fold excess of the displacing protein was taken as an estimate of *k*_off_ along with the standard error of the fit to a single exponential function.

One caveat with ϕ-value analysis is the assumption that ground states are not affected by mutation. Hence, the strategy of using conservative deletion mutations is important (33). For example, mutation to a larger residue is not considered conservative and one of the Trp variants that we tested, NCBD_D/P_^S2078W^, displayed a very low *k*_on_ (3.5 μm^−1^s^−1^) and a 2-fold lower *k*_off_ than the other NCBD variants (Fig. S1). We can only speculate about the structural basis for this result, but because the residue is situated in the loop between Nα1 and Nα2, it might either lock the two helices in relation to each other or flip over, cover the binding groove, and thus block access for CID. In either case we have a clear effect on the ground state. The problem of ground-state changes may be particularly relevant for IDPs, because they are more malleable in terms of structural changes to accommodate binding partners. It is beyond the scope of this study to perform extensive structure determination of all site-directed mutants, but we recorded HSQC spectra for one complex containing a typical deletion mutation and an intermediate ϕ-value, NCBD_D/P_^L2067A^ with CID_1R_^ML^ (Fig. S4, *A* and *B*). The spectra were perturbed by the mutation, with a less marked effect for CID_1R_^ML^. Considering that the amide proton and nitrogen resonances are very sensitive, together with our CD data, the spectra suggest that the structure of the complex between CID_1R_^ML^ and NCBD_D/P_^L2067A^ is compact, stable, and possibly similar to the complex between CID_1R_^ML^ and NCBD_D/P_^pWT^.

**Figure 4.**
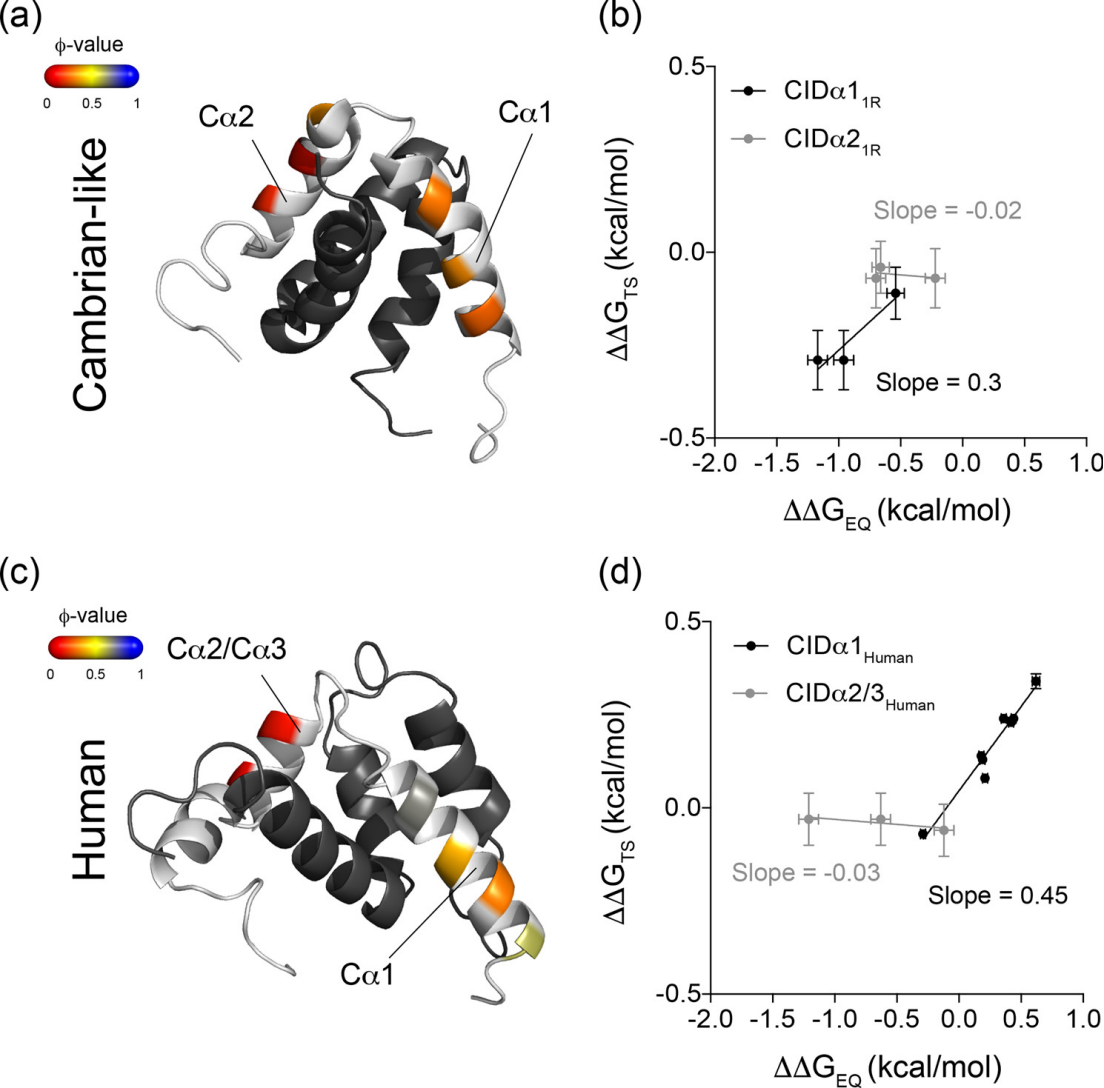
**ϕ-values of helix formation in CID in the Cambrian-like and human complex.** Ala → Gly mutations in surface-exposed positions in the helices of CID_Human_ and CID_1R_^ML^ were introduced and the kinetic parameters for these helix-modulating mutations were obtained in stopped-flow kinetic experiments. The dissociation rate constants were obtained in displacement experiments if *k*_off_ was less than ≈ 30 s^−1^ or otherwise from binding experiments. Using the kinetic parameters (*k*_on_ and *k*_off_), ΔΔG in the transition state (ΔΔG_TS_) and in the bound state (ΔΔG_EQ_) was calculated for each mutant. The experimental conditions were 20 mm sodium phosphate, pH 7.4, and 150 mm NaCl, and the measurements were recorded at 4 °C. *a*, ϕ-values for helix-modulating mutations in helix 1 (Cα1) and helix 2 (Cα2) of CID_1R_^ML^ mapped onto the structure of the Cambrian-like protein complex (PDB entry 6ES5; Dataset S1*A*). *b*, Brønsted plot for the same helix-modulating mutations in CID_1R_^ML^ in the Cambrian-like complex (Dataset S1*B*). The data were fitted with linear regression, yielding slopes of 0.3 ± 0.1 (Cα1) and −0.02 ± 0.04 (Cα2). The *error bars* denote propagated standard errors. *c*, ϕ-values for helix-modulating mutations in helix 1 (Cα1) and helix 2/3 (Cα2/3) of CID in the human complex mapped onto the structure of the complex (PDB entry 1KBH; Dataset S1*D*). *d*, Brønsted plot for helix-modulating mutations in helix 1 (Cα1) and helix 2/3 (Cα2/3) of human CID in complex with human NCBD (Dataset S1*C*). Linear regression analysis yielded slopes of 0.45 ± 0.04 (Cα1) and −0.03 ± 0.02 (Cα2/3). The *error bars* show the propagated standard errors.

### The transition state of the Cambrian-like complex is more native-like than the extant human complex

First, we assessed interactions formed by hydrophobic side chains in the binding interface of the ancestral Cambrian-like complex by computing ϕ-values for each mutant (Table 2). The resulting ϕ-values were mainly in the intermediate category ranging between 0.3–0.6 (Fig. 2*a*). This result was in contrast with previously published low ϕ-values for the human complex (21), which ranged between 0–0.3 for similar conservative deletion mutations (Fig. 2*b*). A notable exception was the CID_Human_^L1055A^ variant, which displayed a ϕ-value of 0.85. This residue is located in the first α-helix, which is transiently populated in the free state of CID and forms many native contacts with NCBD in the transition state (23, 34). The CID_1R_^L1055A^ in the Cambrian-like complex displayed a similarly high ϕ-value (0.83), suggesting that this region forms a conserved nucleus for the coupled binding and folding of CID/NCBD. Further comparison between ϕ-values in the Cambrian-like and human complexes at a site-by-site basis shows that three mutations in particular, NCBD^L2067A^, NCBD^L2087A^, and NCBD^L2096A^, displayed large differences in ϕ-values in the respective complex, with the Cambrian-like complex always showing higher ϕ-values. The differences suggest rearrangements of native contacts in the transition state, resulting in a more disordered transition state for the human complex (Fig. 2*c*).

Accordingly, MD simulations showed that the Cambrian-like TS, as compared with the human one, is significantly more compact (as judged by gyration radius, Fig. 3*e*) and less heterogeneous (as judged by pairwise root mean square deviation, Fig. 3*d*), supporting the idea that the TS for formation of the Cambrian-like complex is more native-like (Video S1). The higher NCBD ϕ-values (Table S1 and Fig. 2*c*) measured for the ancestral complex resulted in differences in both the NCBD secondary/tertiary structure and the intermolecular interactions in the TS. In the ancestral TS, the NCBD helix Nα3 is totally unfolded (consistent with its lower helicity also in the native state) (28), whereas helices Nα1 and Nα2 are well-formed and maintain a native-like relative orientation with numerous Nα1-Nα2 contacts (Fig. 3*b*, *box 1*, and Fig. 3*c*). On the other hand, in the human TS, Nα1 and Nα2 show lower helical content and less frequent contacts. The main intermolecular contacts are conserved in human and Cambrian-like TS: in both the cases we observed a stable hydrophobic core (Fig. 3*b*, *box 2*) involving Cα1 (via residues Leu-1052 and Leu-1055, which display high ϕ-value in both complexes) and Nα1 (via residues Leu-2071, Leu-2074, and Lys-2075) but with a slightly different orientation of the two helices. A second relevant interacting region involves the residues of the unstructured CID helices Cα2-Cα3, which contact NCBD in both TS ensembles. In the ancestral complex, hydrophobic residues of the Nα2 helix are preferred, whereas in the human TS the interactions are more dispersed, involving also the longer and structured helix Nα3 (Fig. 3*b*, *box 3*). The identified interactions are highly native-like in the case of the ancestral complex (Fig. S5) as compared with the human one (23), where a higher number of transient contacts is observed (Fig. S6). We note that the Brønsted plot, where ΔΔG_TS_ is plotted *versus* ΔΔG_EQ_, appears more scattered for the Cambrian-like complex as compared with the human complex and previously characterized IDP systems. A salient feature of a nucleation-condensation mechanism in protein folding, where all noncovalent interactions form cooperatively, is a clear linear dependence of the Brønsted plot, whereas a scattered Brønsted plot suggests a less cooperative binding mechanism with, for example, pre-formed structure. However, the narrow range of ΔΔG_EQ_ values for Cambrian-like NCBD/CID precluded a conclusive comparison of the scatter in the Brønsted plots of the human and Cambrian-like complex.

**Figure 5.**
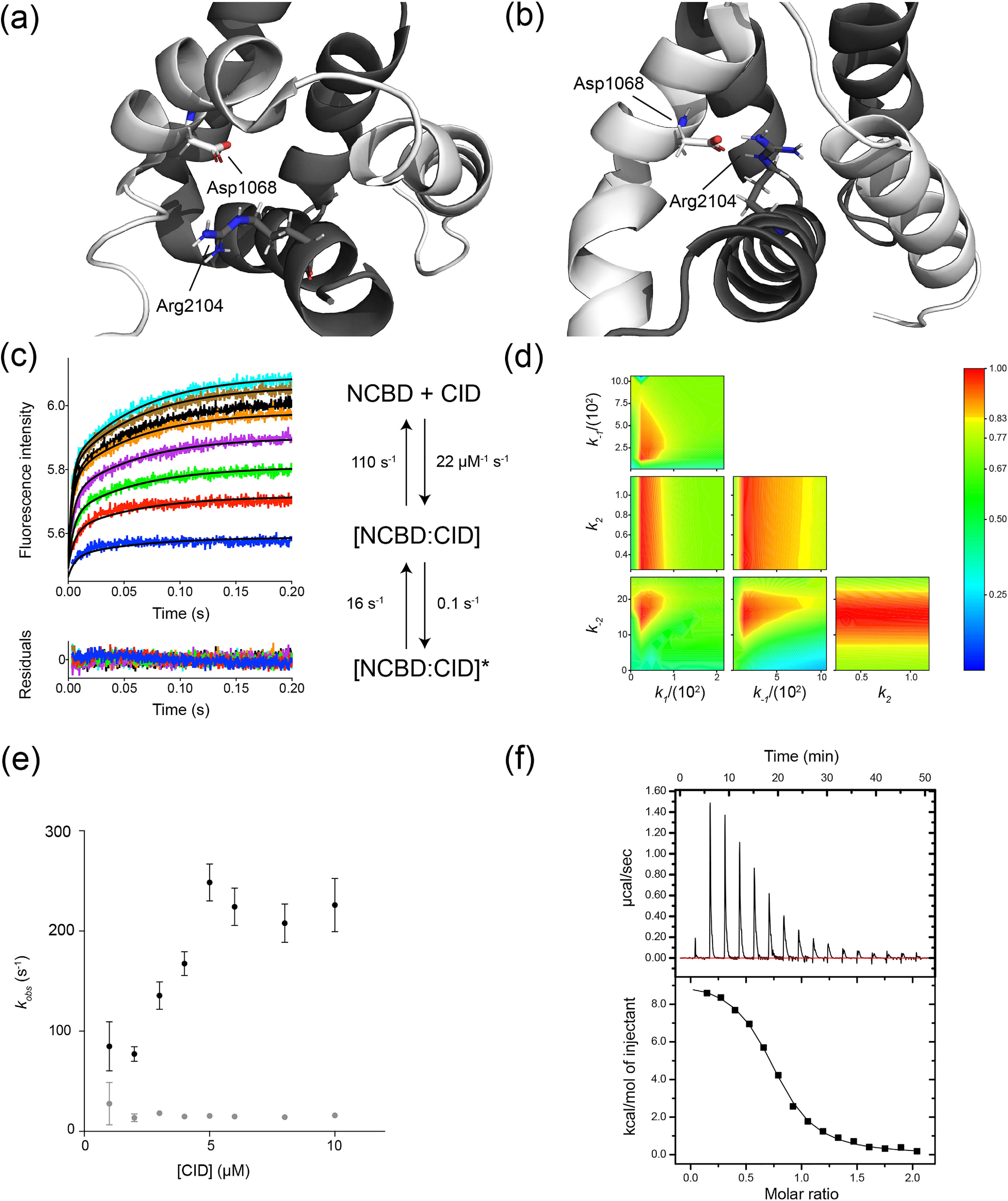
**Mutation of a conserved salt-bridge in the Cambrian-like complex results in population of a minor bound state.** The structure of (*a*) the Cambrian-like (PDB entry 6ES5) and (*b*) the human NCBD/CID complex with Arg-2104 and Asp-1068 forming the salt-bridge highlighted as stick model. NCBD is in *dark gray* and CID in *light gray*. *c*, stopped-flow kinetic traces were fitted globally to an induced-fit model to obtain the microscopic rate constants for each reaction step shown in the scheme. The *black solid lines* represent the best fit to the kinetic traces, and the residuals are shown below the curve. The experiments were performed in 20 mm sodium phosphate, pH 7.4, and 150 mm NaCl at 4 °C. *d*, the confidence contour plot shows the variation in χ^2^ as two parameters are systematically varied while the rest of the parameters are allowed to float, which can reveal covariation between parameters in a model. The color denotes the χ^2^/χ^2^_min_ value according to the *scale bar* to the right. Here, the confidence contour plot showed that *k_2_* was poorly defined. The *yellow boundary* represents a cutoff in χ^2^/χ^2^_min_ of 0.8. *e*, the stopped-flow kinetic traces were fitted to a double exponential function to extract *k*_obs_ values, which were plotted against the concentration of CID_1R_^D1068A^ (Dataset S1*E*). The trends in the *k*_obs_ values suggest one fast linear phase (*black dots*) which reports on binding and one slow phase with a constant *k*_obs_ of 15 s^−1^ (*gray dots*). The *error bars* are standard errors from fitting to a double exponential function. *f*, binding of NCBD_D/P_^R2104M^/CID_1R_^D1068A^ monitored by isothermal titration calorimetry in 20 mm sodium phosphate, pH 7.4, and 150 mm NaCl at 4 °C. Fitting to a two-state model yielded a *K_d_* of 5.1 ± 0.3 µm (Dataset S1*F*).

### The mechanism of helix formation in CID has been well-conserved during evolution

The binding reaction of NCBD and CID is associated with a dramatic increase in secondary structure content of CID. Helical propensity of the N-terminal helix of CID correlates positively with affinity for NCBD (32), and modulation of helical propensity is likely an important evolutionary mechanism for tuning affinities of interactions involving IDPs. To investigate native helix formation in the transition state of the Cambrian-like complex, we introduced Ala → Gly mutations at surface-exposed positions in helix 1 of CID_1R_^ML^ (Cα1_1R_) and in helix 2 (Cα2_1R_). The mutants were subjected to binding experiments, and the rate constants were used to compute ϕ-values for each CID variant in complex with NCBD_D/P_^pWT^ (Table 2). The ϕ-values for Cα1_1R_ ranged between 0.2–0.3 and were lower, close to 0.1, for Cα2_1R_ (Fig. 4*a*). Brønsted plots resulted in slopes for Cα1_1R_ and Cα2_1R_ of 0.3 and 0, respectively (Fig. 4*b*). The slope of the Brønsted plot can be regarded as an average ϕ-value and thus suggests around 30 and 0% native helical content of Cα1_1R_ and Cα2_1R_, respectively, in the transition state for the Cambrian-like complex.

To facilitate a direct comparison with the extant human complex, we extended a previously published dataset on helix 1 from human CID (Cα1_Human_) (32) with new Ala → Gly mutations in helix 2 (Cα2_Human_) and helix 3 (Cα3_Human_) (Table 3). The human complex displayed ϕ-values for helix formation in Cα1_Human_ that ranged from 0.3–0.7, whereas all ϕ-values in Cα2_Human_ and Cα3_Human_ were close to 0 (Fig. 4*c*). This resulted in Brønsted plots with slopes of 0.5 for Cα1_Human_ (32) and virtually 0 for Cα2_Human/_Cα3_Human_ (Fig. 4*d*). Thus, in both the Cambrian-like and human complexes, the N-terminal Cα1 plays an important role in forming early intramolecular native secondary structure contacts in the disorder-to-order transition. These data are well-represented by the ancestral TS ensemble. The probability of α-helix content in CID, measured via DSSP (35) and averaged over the whole ensemble, shows that only helix Cα1 is partially formed in the TS, whereas other CID helices are mostly unstructured (Fig. 3*c*). Analogously, in the TS of the human complex only helix Cα1 was folded, overall supporting the importance of Cα1 formation for NCBD binding.

**Table 3:** **Rate constants for helix-modulating mutations in helix 2 and helix 3 of human CID** The rate constants were obtained in fluorescence-monitored stopped-flow kinetic experiments (Dataset S1*D*). The experimental conditions were 20 mm sodium phosphate buffer, pH 7.4, and 150 mm NaCl, and the experiments were performed at 4 °C. The errors are standard errors from global fitting to a simple two-state model. All experiments were performed once.

NCBD_Human_ variant	CID_Human_ variant	*k*_on_ (µm^−1^ s^−1^)	*k*_off_ (s^−1^)	Type of mutation	*K_d_* (µm)	ϕ-value
pWT	WT	25.0 ± 2.5	2.66 ± 0.01*^a^*	-	0.11 ± 0.01	-
pWT	E1065A	24.9 ± 2.5	2.59 ± 0.01*^a^*	Helix-modulating Cα2	0.10 ± 0.01	-
pWT	E1065G	23.7 ± 2.4	7.8 ± 0.1*^a^*	Helix-modulating Cα2	0.33 ± 0.03	0.04 ± 0.12
pWT	R1069A	27.7 ± 2.8	8.8 ± 0.1*^a^*	Helix-modulating Cα2	0.32 ± 0.03	−0.09 ± 0.13
pWT	R1069G	26.4 ± 2.6	76 ± 2	Helix-modulating Cα2	2.9 ± 0.3	0.02 ± 0.06
pWT	E1075A	26.5 ± 2.7	3.12 ± 0.06*^a^*	Helix-modulating Cα3	0.12 ± 0.01	-
pWT	E1075G	23.7 ± 2.4	3.44 ± 0.02*^a^*	Helix-modulating Cα3	0.15 ± 0.01	-

*^a^* The dissociation rate constant (*k*_off_) was determined in a separate displacement experiment. The value of *k*_off_ was estimated from a measurement with 20-fold excess of unlabeled NCBD, and the errors are the standard error from the fit to a single exponential function.

We note that according to Brønsted plots, Cα1_Human_ has a slightly higher helical content in the transition state compared with Cα1_1R_, consistent with a higher helical propensity for Cα1_Human_ than for Cα1_1R_, as suggested by predictions using AGADIR (4). We further note that the A1075G mutation in Cα3_Human_ did not display a large effect on *K_d_*, which precluded a reliable estimation of a ϕ-value. This could indicate either that Cα3_Human_ contributes little to the stability of the bound complex or that the Ala → Gly substitution at this position promotes an alternative conformation that binds with equal affinity as the WT protein. Similarly, Val-1077 → Ala in Cα3_Human_ was shown previously to have a small positive effect on the affinity for NCBD (21), suggesting structural rearrangement in the bound state.

### Role of a conserved and buried salt-bridge in the Cambrian-like complex

Long-range electrostatic interactions promote association of proteins and play a major role in IDPs. Mutation of a conserved salt-bridge between Arg-2104 in NCBD and Asp-1068 in CID was previously shown to display large effects on the kinetics of complex formation for human NCBD/CID, both in terms of a 10-fold reduction in *k*_on_ and with the occurrence of a new kinetic phase (*k*_obs_ ≈ 15–20 s^−1^). Analysis of the kinetic data favored an induced-fit model, and thus a conformational change after binding (24, 36, 37). We generated the protein variants NCBD_D/P_^R2104M^ and CID_1R_^D1068A^ to assess the role of this salt-bridge in the ancestral Cambrian-like complex.

NCBD_D/P_^R2104M^ and CID_1R_^D1068A^ displayed clear biphasic kinetic traces in the stopped-flow experiments, similarly to experiments with the corresponding mutants in the human complex (Fig. 5). Fitting of the kinetic data to obtain *k*_obs_ values revealed one concentration-dependent kinetic phase, which increased linearly with CID concentration, and a second kinetic phase, which was constant at *k*_obs_ ≈ 16 s^−1^ over the entire concentration range. Thus, the kinetic dataset for the complex between NCBD_D/P_^R2104M^/CID_1R_^D1068A^ was fitted globally to an induced-fit mechanism to obtain estimates of the microscopic rate constants. The comparison between the Cambrian-like and human complexes revealed that the effect of mutating the buried salt-bridge was much smaller with regard to the association rate constant *k_1_* for the Cambrian-like complex than for the human complex, less than 2-fold *versus* 20-fold, respectively. On the other hand, the slow phase was similar for both the human and ancestral complexes with a *k*_obs_ (= *k_2_* + *k_−2_*) of 15–20 s^−1^. However, global fitting suggested that the alternative conformation of the bound state is only slightly populated for the Cambrian-like complex (*k_−2_≫k_2_*) (Fig. 5). This was corroborated by isothermal titration calorimetry measurements, which showed that the overall *K_d_* (5.1 ± 0.3 μm) is highly consistent with *k_−1_*/*k_1_* (4.8 μm). Interestingly, whereas the salt-bridge is significantly populated in the native-state simulations, it is not populated in either the Cambrian-like or the human TS ensemble, suggesting that the formation of this interaction is not relevant for the initial recognition. Nonetheless, these residues promote the association of the human complex most likely via unspecific long-range interactions.

Furthermore, we mutated Asp-1053 in CID_1R_^ML^ to Ala, to assess potential salt-bridge formation between this residue and Arg-2104 in NCBD_D/P_^ML^. The complex between NCBD_D/P_^R2104M^ and CID_1R_^D1053A^ was very destabilized, and the kinetic phase that reported on the binding event was too fast for the stopped-flow instrument. However, the concentration-independent kinetic phase was detected (*k*_obs_ ≈ 20–40 s^−1^). Single charge mutations cannot be considered conservative because they may result in unpaired charges in or close to hydrophobic interfaces. Any effects from such mutations may also be due to nonspecific charge-charge attraction or repulsion. (The overall charge of NCBD_D/P_ is positive and CID_1R_ is negative.) Nevertheless, we report kinetic data for such single mutants. Interestingly, CID_1R_^D1068A^ displayed a negative ϕ-value (∼−0.4, Table 2 and Table 1) because of an increase in both *k*_on_ and *k*_off_ upon mutation, suggesting that Asp-1068 makes a nonfavorable interaction in the transition state. This is consistent with the small effect on *k*_on_ for NCBD_D/P_^R2104M^/CID_1R_^D1068A^, which might result from opposing effects on *k*_on_ by the respective mutation. The other Asp mutant, CID_1R_^D1053A^, also displayed an increase in *k*_on_ but a positive high ϕ-value. Thus, Asp-1053 forms nonfavorable interactions both in the transition state and in the native state of the complex. The NCBD_D/P_^K2075M^ variant gives a high ϕ-value, suggesting a native interaction in the transition state. Although Asp-1053 is in the vicinity of Lys-2075, the coupling free energy between them is low (0.17 kcal/mol) and it is not clear what interactions these residues make in the native complex. All surface charge mutations had little effect on kinetics or yielded low ϕ-values, suggesting that overall charge plays a minor role in the association of NCBD_D/P_ and CID_1R_ (Table 2) and similarly for human NCBD/CID (Table 3).

In agreement with these data, simulations supported the idea that hydrophobic interactions are more relevant than electrostatic contacts in the TS for formation of the ancestral complex. In fact, no stable salt-bridges or hydrogen bonds were observed, with Arg-2104 contacting different polar residues only in a transient manner. Also, the high ϕ-value of the NCBD_D/P_^K2075M^ variant can be explained by the ability of Lys-2075 to engage in hydrophobic, rather than polar, interactions stabilizing the native-like hydrophobic core formed by helices Cα1-Nα1 (Fig. 3*b* and Fig. S5).

## Discussion

The higher prevalence of IDPs among eukaryotes as compared with prokaryotes suggests that these proteins have played an important role in the evolution of complex multicellular organisms (38, 39). IDPs often participate in regulatory functions in the cell by engaging in complex interaction networks that fine-tune cellular responses to environmental cues (40). One feature common to many IDPs, and which has likely contributed to their abundance in regulatory functions, is the ability to interact specifically with several partners that are competing for binding (41). NCBD is an archetype example of such a disordered protein interaction domain that has evolved to bind several cellular targets, including transcription factors and transcriptional coregulators (18, 19, 42). Every time a new partner was included in the repertoire, NCBD somehow adapted its affinity for the new ligand while maintaining affinity for already established one(s), as occurred around 450–500 million years ago, when the interaction between NCBD and CID was established (4). On a molecular level, it is intriguing how such multi-partner protein domains evolve. In the present study, we have extended our structural studies (28) and investigated the evolution of the binding mechanism using site-directed mutagenesis, ϕ-value analysis, and restrained MD simulations to shed light on changes occurring at the molecular level when the low-affinity Cambrian-like NCBD evolved higher affinity for its protein ligand CID.

Recent works suggest that IDPs can adopt multiple strategies for recognizing their partners. Gianni and co-workers (43) proposed the concept of templated folding, where the folding of the IDP is modulated, or templated, by its binding partner, as shown for cMyb/KIX (44), MLL/KIX (45), and N_TAIL_/XD (46, 47). Similar ideas were put forward by Zhou and co-workers (48) based on experiments on WASP GBD/Cdc42 and formulated in terms of multiple dock-and-coalesce pathways. On the other hand, studies on disordered domains from BH3-only proteins binding to BCL-2 family proteins suggest conservation of ϕ values and a more robust folding mechanism (49). We recently showed by double mutants and simulation that a high plasticity in terms of formation of native hydrophobic interactions in the transition state exists for human NCBD/CID (23) where both partners are very flexible, in agreement with templated folding.

In the present study, by comparing the TSs for formation of human and Cambrian-like NCBD/CID complexes, we demonstrate that, although similar core interacting regions have been conserved throughout evolution, the interaction between the two proteins has evolved from a more ordered ancestral TS to the heterogenous and plastic behavior observed in the human complex. We find that the fraction of CID helical content in the transition state is overall conserved, with intermediate values in Cα1 (slightly higher in human than in Cambrian-like NCBD/CID) and low values in Cα2/3. Conversely, we observe that the transition state of the low-affinity Cambrian-like complex has more native-like features in terms of hydrophobic interactions (higher ϕ-values) as compared with the human one, with clear site-specific differences such as residues Leu-2067, Leu-2087, and Leu-2096 of NCBD. In the ancestral TS, fewer but more-native-like contacts are required to be formed (Fig. S5 and Fig. S6) and proper NCBD tertiary structure (regulating Nα1-Nα2 orientation) is achieved before CID binding. Vice versa, in the TS of human NCBD/CID, numerous transient intermolecular interactions are engaged (Fig. S6), involving a large number of residues of both CID and NCBD.

There is always uncertainty in reconstructed ancient sequences. It is therefore important to assess whether the conclusions are robust to the inevitable errors present in reconstructed ancient sequences. In the present case we have not performed a ϕ-value analysis for alternative reconstructed NCBD or CID variants because of the extensive experimental effort. However, the overall similar transition states of the human and ancient complexes suggest that the overall mechanism is robust and that further point mutations would not dramatically change this picture. Furthermore, the five ϕ-values determined with an alternative Trp probe (Trp-2067) were very similar to those with Trp-2073 (Table 1), also demonstrating that the mechanism is robust to mutation. Finally, the NCBD/CID affinity is relatively constant across the deuterostome animal kingdom (Fig. 1) despite several differences in the primary structure (4, 27), and it is therefore likely that the binding mechanism is also conserved.

In the homeodomain family of proteins, a spectrum of folding mechanisms was previously observed (50) ranging from nucleation-condensation to diffusion-collision (51). Furthermore, it has been suggested that the two mechanisms can be related to the balance between hydrophobic and electrostatic interactions (52). Mutational studies on IDPs (21, 44, 45, 53–58) are more or less consistent with apparent two-state kinetics and the nucleation-condensation mechanism of globular proteins (59), *i.e.* cooperative, simultaneous formation of all noncovalent interactions around one well-defined core. Salient features of this mechanism are linear Brønsted plots and fractional ϕ-values. One alternative mechanism would be independently folding structural elements, which dock to form the tertiary structure as formulated in the diffusion-collision model (51). In such scenario, Brønsted plots would be more scattered and the ϕ-values would be both low and high, clustered in structurally contiguous contexts, and even separated into two or more folding nuclei. Our comparison of secondary and tertiary structure formation in human *versus* Cambrian-like NCBD/CID is therefore interesting because it shows that the folding of certain elements of secondary structure can be distinct from others and that they may or may not be part of an extended folding nucleus. The Brønsted plot for Cambrian-like NCBD/CID shows a larger scatter than that for human NCBD/CID (Fig. 2*d*). Whereas Cα1 of both CID_1R_^ML^ and human CID displays fractional ϕ-values, Cα2/3 have ϕ-values of zero (Fig. 4). Thus, Cα1 may function as a well-defined folding nucleus around which remaining structure condensates, as observed for high-affinity human NCBD. However, Cα1 may also be part of a more extended folding nucleus together with hydrophobic tertiary interactions as in low-affinity Cambrian-like NCBD/CID (Figs. 2–4) but not to the extent that we define it as two separate folding nuclei. The Cambrian-like NCBD/CID shows therefore an intermediate behavior between nucleation-condensation and diffusion-collision mechanism, which shifted toward nucleation-condensation during evolution. A similar diffusion-collision-like mechanism was recently found in the interaction between disordered YAP and TEAD (60). However, three other IDP interactions with more than one helical segment, Hif-1α CAD (58), TAD-STAT2 (57), and pKID (61) (all binding to KIX), do not display this behavior but show mainly low ϕ-values (<0.2) with only one or a few higher ones. Thus, so far, nucleation-condensation appears more prevalent for globular protein domains (62) and for IDPs in disorder-to-order transitions. It will be interesting to see whether other IDPs with several secondary structure elements display any distinct distribution of ϕ-values.

## Experimental procedures

### Ancestral and human protein sequences

The reconstruction of ancestral sequences of NCBD (from the CREBBP/p300 protein family) and CID (from the NCOA/p160/SRC protein family) has been described in detail before (4). Briefly, protein sequences in these families from various phyla were aligned and the ancestral protein sequences of NCBD and CID were predicted using an ML method. The ML ancestral protein variants of NCBD and CID, NCBD_D/P_^ML^ and CID_1R_^ML^, were used as WTs in this study. The human NCBD protein was composed of residues 2058–2116 from human CREBBP (UniProt ID: Q92793) and the human CID domain was composed of residues 1018–1088 from human NCOA3/ACTR (UniProt ID: Q9Y6Q9), in accordance with previous studies on the human protein domains (21, 23, 24). The reconstructed ancestral sequences of NCBD and CID were shortened to contain only the evolutionarily more well-conserved regions that form a well-defined structure upon association with the other domain. Thus, the ancestral NCBD variant was composed of residues corresponding to 2062–2109 in human CREBBP and the ancestral CID variant was composed of residues corresponding to 1040–1081 in human NCOA3/ACTR.

### Cloning and mutagenesis

The cDNA sequences for the protein variants used in the study were purchased from GenScript and the proteins were N-terminally tagged with a His_6_-Lipo domain. The mutants were generated using a whole plasmid PCR method. The primers were typically two complementary 33-mer oligonucleotides with mismatching bases at the site of the mutation, which were flanked on each side by 15 complementary bases. The annealing temperature in the PCR reactions was between 55–65 °C and the reactions were run for 20 cycles. The products were transformed into *Escherichia coli* XL-1 Blue Competent Cells and selected on LB agar plates with 100 μg/ml ampicillin. The plasmids were purified using the PureYield^TM^ Plasmid Miniprep System (Promega).

### Protein expression and purification

The plasmids encoding the protein constructs were transformed into *E. coli* BL-21 DE3 pLysS (Invitrogen) and selected on LB agar plates with 35 μg/ml chloramphenicol and 100 μg/ml ampicillin. Colonies were used to inoculate LB media with 50 μg/ml ampicillin and the cultures were grown at 37 °C to reach *A*_600_ 0.6–0.7 prior to induction with 1 mm isopropyl β-d-1-thiogalactopyranoside and overnight expression at 18 °C. The cells were lysed by sonication and centrifuged at ∼50,000 × *g* to remove cell debris. The lysate was separated on a Ni-Sepharose 6 Fast Flow (GE Healthcare) column using 30 mm Tris-HCl, pH 8.0, and 500 mm NaCl as the binding buffer and 30 mm Tris-HCl, pH 8.0, 500 mm NaCl, and 250 mm imidazole as the elution buffer. The His_6_-Lipo tag was cleaved off using Thrombin (GE Healthcare), and the protein was separated from the cleaved tag using the same column and buffers as described above. Lastly, the protein was separated on a RESOURCE^TM^ reversed-phase chromatography column (GE Healthcare) using a 0–70% acetonitrile gradient. The purity of the protein was verified by the single-peak appearance on the chromatogram or by SDS-PAGE. The identity was verified by MALDI-TOF MS. The fractions containing pure protein were lyophilized and the concentration of the protein was measured by absorption spectrometry at 280 nm for variants that contained a Tyr or Trp residue. For the proteins that lacked a Tyr or Trp residue, absorption at 205 nm was used to estimate the concentration. The extinction coefficient for human CID was previously determined by amino acid analysis to 250,000 m^−1^ cm^−1^ at 205 nm. For the shorter ancestral variants, the extinction coefficient was calculated based on the amino acid sequence (64).

### Design and evaluation of NCBD_D/P_^ML^ Trp variants

To perform fluorescence-monitored stopped-flow kinetic experiments, a fluorescent probe is required. Because both NCBD and CID lack Trp residues, which provide the best sensitivity in fluorescence-monitored experiments, several NCBD_D/P_^ML^ variants with Trp residues introduced at different positions were constructed: NCBD_D/P_^L2067W^, NCBD_D/P_^T2073W^, NCBD_D/P_^S2078W^, NCBD_D/P_^H2107W^, and NCBD_D/P_^Q2108W^. The NCBD_D/P_^Q2108W^ variant corresponds to the NCBD_Human_^Y2108W^ variant, which was used previously as a pseudo-WT in stopped-flow kinetic experiments (21, 23, 24). These NCBD_D/P_^ML^ Trp variants were assessed based on secondary structure content and stability of complex with CID using far-UV CD spectroscopy, and by kinetic and equilibrium parameters from stopped-flow fluorescence spectroscopy and ITC, to find an engineered NCBD_D/P_^ML^ variant with similar biophysical properties to the WT NCBD_D/P_^ML^ (Fig. S1). The NCBD_D/P_^T2073W^ variant displayed the most similar behavior to NCBD_D/P_^ML^. Our data showed that the structural content, complex stability, and affinity of this Trp variant were highly similar to those of NCBD_D/P_^ML^ (Fig. S1). Thus, our data validated the use of NCBD_D/P_^T2073W^ variant as a representative of NCBD_D/P_^ML^, and all stopped-flow kinetic experiments for the ancestral Cambrian-like complex were performed using the pseudo-WT NCBD_D/P_^T2073W^ variant, which we denote NCBD_D/P_^pWT^.

### Stopped-flow spectroscopy and calculation of ϕ-values

The kinetic experiments were conducted using an upgraded SX-17MV stopped-flow spectrofluorometer (Applied Photophysics). The excitation wavelength was set to 280 nm, and the emitted light was detected after passing through a 320-nm long-pass filter. All experiments were performed at 4 °C, and the default buffer for all experiments was 20 mm sodium phosphate, pH 7.4, and 150 mm NaCl. To promote secondary and tertiary structure formation of some structurally destabilized NCBD mutants, the experimental buffer was supplemented with 0.7 m TMAO (Fig. S2, *C* and *D*). Typically, in kinetic experiments the concentration of NCBD was kept constant at 1–2 μm and the concentration of CID was varied between 1–10 μm. Experiments where the concentration of CID was kept constant while NCBD was varied were also performed to check for consistency of the obtained results. In these experiments, CID was held constant at 2 μm and NCBD was varied between 2–10 μm. The kinetic binding curves with NCBD in excess was in good agreement with those using CID in excess, but because the quality of the kinetic traces was better when CID was in excess, these experiments were used to determine the kinetic parameters for the different variants reported in the paper. All experiments were performed using the pseudo-WT NCBD variants, which were the NCBD_D/P_^T2073W^ and NCBD_Human_^Y2108W^ variants. These variants are denoted as NCBD_D/P_^pWT^ and NCBD_Human_^pWT^, respectively.

Each stopped-flow trace consisted of 1000 sampled data points (data points before 0.002 s were removed because of insufficient mixing within this time period). Each kinetic trace is typically an average of 4–10 individual traces (*i.e.* 4–10 technical replicates). When the data were fitted globally, the large number of data points resulted in underestimated standard errors. To correct for this, we determined the error in *k*_on_ based on five biological replicates (*i.e*. performed with different protein batches), which was 10% for the NCBD_D/P_^pWT^/CID_1R_^ML^ complex. This error was then applied on *k*_on_ for all other protein variants.

The dissociation rate constant *k*_off_ can be determined from binding experiments, but the accuracy decreases whenever *k*_obs_ values are much larger than *k*_off_ or when *k*_off_ is very low. In the present study, displacement experiments were performed for protein complexes with *k*_off_ values below 30 s^−1^ in binding experiments. In displacement experiments, an unlabeled NCBD variant (without Trp) was used to displace the different NCBD_D/P_^pWT^ or NCBD_Human_^pWT^ variants from the complexes. The *k*_obs_ value at 20-fold excess of the unlabeled NCBD variant was taken as an estimate of the dissociation rate constant, *k*_off_.

The ϕ-values for each mutant were computed using the rate constants that were obtained in the stopped-flow measurements (*e.g. k*_on_ and *k*_off_) using Eqs. 1–3.

(Equation 1) $$\Delta \Delta G_{\mathrm{TS}}=\mathrm{RT}\;\ln\;\left(k_{on}{}^{\mathrm{mt}}/k_{on}{}^{\mathrm{wt}}\right)$$

(Equation 2) $$\Delta \Delta G_{\mathrm{EQ}}=\mathrm{RT}\;\ln\;\left(K_{d}{}^{\mathrm{wt}}/K_{d}{}^{\mathrm{mt}}\right)$$

(Equation 3) $$\phi -\mathrm{value}=\Delta \Delta G_{\mathrm{TS}}/\Delta \Delta G_{\mathrm{EQ}}$$

### CD spectroscopy

Far-UV CD spectra were acquired using a J-1500 spectrophotometer (JASCO) in 20 mm sodium phosphate buffer, pH 7.4, and 150 mm NaCl at 4 °C. The bandwidth was 1 nm, the scanning speed was 50 nm/min, and the data pitch was 1 nm. The protein concentrations were between 20–40 μm for all protein variants and each spectrum was typically an average of 2–3 individual spectra. The thermal denaturation experiments of the protein complexes were performed by monitoring the CD signal of 20 μm NCBD in complex with 20 μm CID at 222 nm in the same experimental buffer as above and over a temperature range of 4–95 °C. For these experiments, the heating speed was 1 °C/min with 5 s waiting time at each data point and data were acquired every 1 °C.

### NMR spectroscopy

NMR samples were prepared by mixing the labeled NCBD or CID solution with excess amount of unlabeled CID or NCBD solution, followed by lyophilization and rehydration. The final samples had a labeled NCBD or CID concentration of ∼0.5 mm and a phosphate buffer concentration of 20 mm at pH 7. During dissolution, 0.01% NaN_3_ and 10% D_2_O were added. All NMR spectra were recorded at 25 °C on a 600 MHz Bruker Avance Neo NMR spectrometer equipped with a TCI CryoProbe. The ^1^H-^15^N HSQC spectra were collected with 2048 data points in ω_2_/^1^H dimension and 256 data points in ω_1_/^15^N dimension; four or eight scans were taken. All spectra were processed with TopSpin 3.2 and analyzed with Sparky 3.115 (65). During this analysis, a downfield shift of 1.0 ppm in ^15^N dimension and a downfield shift of 0.13 ppm in ^1^H dimension were specifically applied to the ppm scale for ^15^N HSQC spectrum of unlabeled CID_1R_^ML^ bound to ^15^N-labeled NCBD_D/P_^pWT^.

### Isothermal titration calorimetry

Isothermal titration calorimetry measurements were performed at 25 °C in a MicroCal iTC_200_ System (GE Healthcare). The proteins were dialyzed simultaneously in the same experimental buffer (20 mm sodium phosphate, pH 7.4, and 150 mm NaCl) to reduce buffer mismatch. The concentration of NCBD in the cell was 12–50 μm (depending on variant) and the concentration of CID in the syringe was between 120–500 μm, depending on NCBD concentration, such that a 1:2 stoichiometry was achieved at the end of each experiment. The data were fitted using the built-in software to a two-state binding model.

### Data analysis using numerical integration

The stopped-flow kinetic datasets were fitted using the KinTek Explorer software (KinTek Corporation) (66, 67). The software employs numerical integration to simulate and fit reaction profiles directly to a mechanistic model. Scaling factors were used to correct for small fluctuations in lamp intensity and errors in concentration, but they were generally close to 1. In cases where the signal-to-noise in the obtained data were low, scaling factors were not applied. For some more-than-one-step models, two-dimensional confidence contour plots were computed to assess confidence limits for each parameter and covariation between parameters. An estimated real time zero of the stopped-flow instrument of −1.25 ms was used to adjust the timeline to obtain correct kinetic amplitudes. The fitted data were exported and graphs were created in GraphPad Prism version 6.0 (GraphPad Software).

### MD simulations of the transition state ensembles

The transition state for formation of the human complex was previously determined by means of ϕ-value restrained molecular dynamics simulations (23). Here, the same procedure was followed to determine the TS of the ancestral CID-NCBD complex. The simulations were performed with GROMACS 2018 (68) and the PLUMED2 software (69), using the Amber03w force field (70) and the TIP4P/2005 water model (71). The initial conformation was taken from available PDB structure (6ES5) (28) and modified with Pymol (72) to account for the T2073W mutation. The structure was solvated with ∼6700/16800 water molecules (for native-state and TS simulations, respectively), neutralized, minimized, and equilibrated at 278 K using the Berendsen thermostat (73). Production simulations were run in the canonical ensemble, thermosetting the system using the Bussi thermostat (74); bonds involving hydrogens were constrained with the LINCS algorithm (75), electrostatic was treated by using the particle mesh Ewald scheme (76) with a short-range cutoff of 0.9 nm, and van der Waals interaction cutoff was set to 0.9 nm.

A reference native-state simulation, at 278 K, was performed to determine native contacts. First, we ran a 40-ns restrained simulation to enforce agreement with atomic intermolecular upper distances previously determined from NMR experiments (28): to this aim, lower wall restraints were applied on the NOE-converted distances. Subsequently, an unrestrained 280-ns simulation was performed and the last 200 ns were used to determine native contacts; given two residues that are not nearest neighbors, native contacts are defined as the number of heavy side-chain atoms within 0.6 nm in at least 50% of the frames.

The TS ensemble of the ancestral complex was determined via ϕ-value restrained MD simulations, following a standard procedure based on the interpretation of ϕ-value analysis in terms of fraction of native contacts (23, 77, 78). Herein, restraints (in the form of a pseudo-energy term accounting for the square distance between experimental and simulated ϕ-values) are added to the force field to maximize the agreement with the experimental data; the underlying hypothesis is that structures reproducing all the measured ϕ-values are good representations of the TS. From each conformation the ϕ-value for a residue is back-calculated as the fraction of the native contact (determined from the native-state simulation) that it makes, implying that only ϕ-values between 0 and 1 can be used as restraints. In total, we included 11 ϕ-values in this range, all based on single conservative point mutations. Mutations involving charged amino acids (namely, K2075M, involved in intermolecular interactions, and the Ala → Gly substitutions at positions Asp-1050 and Arg-1069, probing the helical content of CID helices Cα1 and Cα2, respectively) were excluded; we however verified that the structural ensemble obtained could provide a consistent interpretation of the associated ϕ-values. A list of the ϕ-values used in the ancestral and human TS simulations is reported in Table S1. The TS ensemble was generated using simulated annealing, performing 1334 annealing cycles, each 150 ps long, in which the temperature was varied between 278 and 378 K, for a total simulation time of 200 ns. The TS was determined using only the structures sampled at the reference temperature of 278 K in the last 150 ns of simulation, resulting in an ensemble of ∼5400 conformations. All the input files needed to perform the TS MD simulation are available on the PLUMED-NEST repository (79), as plumID: 2020-021.

## Data availability

All data are contained within this article and in the supporting information. All kinetic and thermodynamic data and calculations of ϕ-values are compiled in an excel file provided as Dataset S1.

## Supplementary Material

Supporting Information
