# Supplementary material for: Mapping the transition state for a binding reaction between ancient intrinsically disordered proteins
Source: J Biol Chem. 2020 Oct 16;295(51):17698–712. doi: 10.1074/jbc.RA120.015645 (PMC7762952; doi:10.1074/jbc.RA120.015645)
Supplement: Supporting Information [file supp_295_51_17698__index.html]

Mapping the transition state for a binding reaction between ancient intrinsically disordered proteins — Evolution of a binding transition state — Mapping the transition state for a binding reaction between ancient intrinsically disordered proteins — Evolution of a binding transition state — Supporting Information 

# Mapping the transition state for a binding reaction between ancient intrinsically disordered proteins

## Supporting Information

- Supporting Video S1. - Supporting Video S1.
- Supporting Information (to be published online) - Excel file containing all kinetic and thermodynamic parameters and calculations.
- Supporting Information (to be published online) - Supporting table and figures
