# Supplementary material for: Mapping the transition state for a binding reaction between ancient intrinsically disordered proteins
Source: J Biol Chem. 2020 Oct 16;295(51):17698–712. doi: 10.1074/jbc.RA120.015645 (PMC7762952; doi:10.1074/jbc.RA120.015645)
Supplement: Supporting Information [file supp_RA120.015645_162930_2_supp_612907_q18y1x.pdf]

Supporting information

Evolution of increased heterogeneity in the binding transition state for intrinsically disordered proteins

Elin Karlsson[1], Cristina Paissoni[2], Amanda M. Erkelens[1,3], Zeinab A. Tehranizadeh[1,4], Frieda A. Sorgenfrei[1,5], Eva Andersson[1], Weihua Ye[1], Carlo Camilloni[2,]*, and Per Jemth[1,]*

[1]Department of Medical Biochemistry and Microbiology, Uppsala University, BMC Box 582, SE-75123 Uppsala, Sweden.
[2]Dipartimento di Bioscienze, Università degli Studi di Milano, 20133 Milano, Italy.
[3]Present address: Department of Chemistry, Leiden University, Leiden, Netherlands.
[4]Department of Medicinal Chemistry, School of Pharmacy, Mashhad University of Medical Sciences, Mashhad, Iran.
[5]Present address: Department of Chemistry, Institute of Organic and Bioorganic Chemistry, University of Graz, Heinrichstraße 28, 8010 Graz, Austria.

*Correspondence to:
Per Jemth, Per.Jemth@imbim.uu.se, phone: +46-18-471 4557
Carlo Camilloni, carlo.camilloni@unimi.it, phone: + 39-02-503 14918

Running title: Evolution of a binding transition state

Keywords: Intrinsically disordered proteins, phi value analysis, transition state, protein evolution, coupled binding and folding

Table S1. ϕ-values used in ancestral and human TS simulations. List of ϕ-values used as restrains in MD simulations for the determination of the ancestral and human TS ensembles.

Protein	ResID	Human Mutation	Human ϕ-value	Ancestral Mutation	Ancestral ϕ-value
NCBD	2067	L2067A	0.00 ± 0.09	L2067A	0.42 ± 0.08
NCBD	2070	L2070A	0.20 ± 0.04	L2070A	0.44 ± 0.11
NCBD	2074	L2074A	0.19 ± 0.03	/	/
NCBD	2086	V2086A	0.00 ± 0.02	/	/
NCBD	2087	L2087A	0.14 ± 0.03	L2087A	0.91 ± 0.23
NCBD	2096	L2096A	0.16 ± 0.03	L2096A	0.81 ± 0.34
NCBD	2099	A2099G	0.23 ± 0.04	A2099G	0.06 ± 0.05
NCBD	2109	V2109A	0.22 ± 0.09	/	/
CID	1043	S1043M	0.66 ± 0.09	/	/
CID	1047	A1047G	0.24 ± 0.05	A1047G	0.21 ± 0.13
CID	1048	L1048A	0.27 ± 0.02	L1048A	0.36 ± 0.06
CID	1049	L1049A	0.17 ± 0.02	L1049A	0.31 ± 0.09
CID	1050	D1050E	0.35 ± 0.07	/	/
CID	1054	T1054Q	0.74 ± 0.12	S1054G	0.24 ± 0.07
CID	1055	L1055A	0.85 ± 0.10	L1055A	0.83 ± 0.18
CID	1056	L1056A	0.07 ± 0.02	/	/
CID	1062	/	/	M1062G	0.31 ± 0.37
CID	1064	L1064A	0.06 ± 0.02		/
CID	1065	/	/	A1065G	0.05 ± 0.11
CID	1067	I1067V	0.16 ± 0.02	I1067V	0.23 ± 0.08
CID	1071	L1071A	0.14 ± 0.03	/	/
CID	1073	I1073V	0.15 ± 0.05	/	/
CID	1077	V1077A	0.00 ± 0.13	/	/

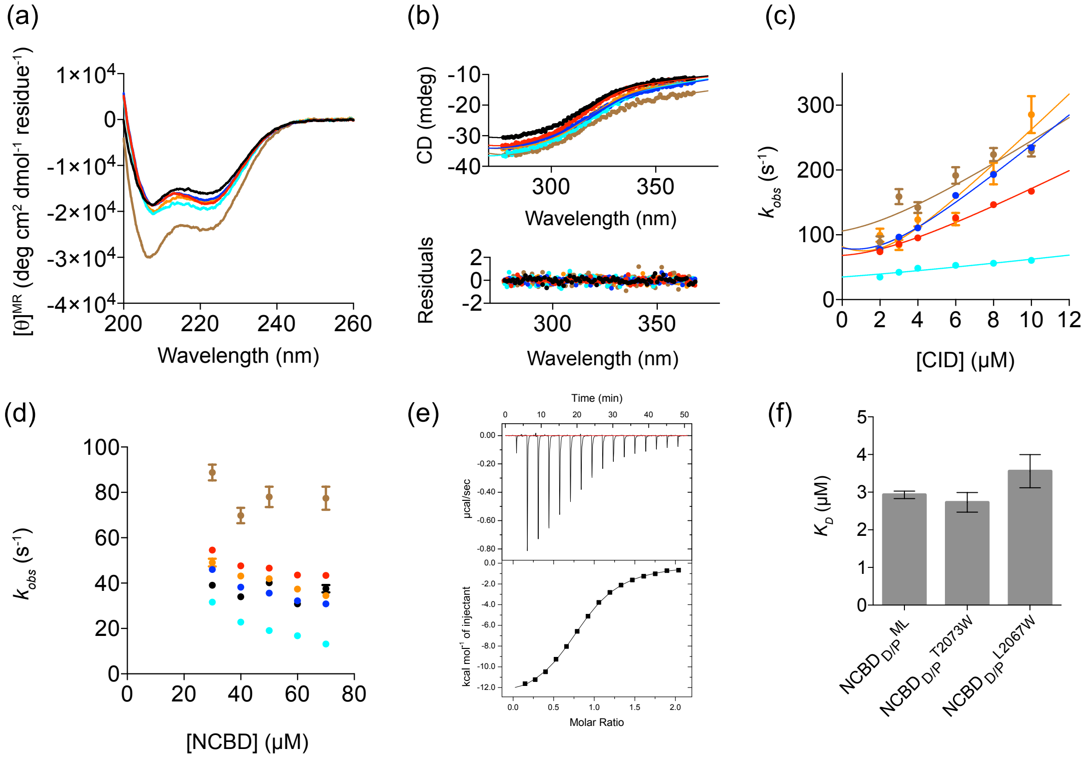

$NCBD_{D/P}$ variant	T_M (K)	k_{on} (μM^{-1} s^{-1}) Stopped-flow	K_d (μM) ITC
ML	311 ± 1	-	2.9 ± 0.1
L2067W	311 ± 1	15 ± 1	3.6 ± 0.4
T2073W	314 ± 1	24 ± 1	2.7 ± 0.3
S2078W	320 ± 2	3.5 ± 0.7	-
H2107W	309 ± 3	19 ± 5	-
Q2108W	313 ± 1	28 ± 4	-

Figure S1. Structural, thermodynamic and kinetic properties of different $NCBD_{D/P}^{ML}$ Trp variants. For all datasets, the color coding is the following: $NCBD_{D/P}^{ML}$ (black), $NCBD_{D/P}^{L2067W}$ (red), $NCBD_{D/P}^{T2073W}$ (blue), $NCBD_{D/P}^{S2078W}$ (cyan), $NCBD_{D/P}^{H2107W}$ (brown) and $NCBD_{D/P}^{Q2108W}$ (orange). The experiments were conducted in 20 mM sodium phosphate pH 7.4, 150 mM NaCl at 4°C unless otherwise stated. All experiments were performed once. (a) CD spectra for $NCBD_{D/P}^{ML}$ and the different $NCBD_{D/P}^{ML}$ Trp variants. (b) Thermal stability of different $NCBD_{D/P}^{ML}$ Trp variants in complex with CID_{1R}^{ML}. The CD signal at 222 nm was used to monitor complex dissociation and the temperature interval was 4-95 °C. The data were fitted to a two-state model (solid line, residuals in lower panel) in order to obtain estimates of the thermal denaturation midpoint of the respective NCBD/CID complex (Supporting Dataset S1g). (c) Observed rate constants (k_{obs}) from stopped flow binding experiments for $NCBD_{D/P}^{ML}$ Trp variants and CID_{1R}^{ML}. The concentration of NCBD was held constant at 2 μM and the concentration of CID was varied from 2-10 μM. The datasets were fitted to a two-state function for bimolecular association to obtain estimates of the association rate constants (k_{on}; Supporting Dataset S1h) (1). (d) Observed rate constants from stopped flow displacement experiments for $NCBD_{D/P}^{ML}$ Trp variants and $NCBD_{D/P}^{ML}$ in complex with CID_{1R}^{ML} (Supporting Dataset S1i). The $NCBD_{D/P}^{ML}$ variant was used to displace the different $NCBD_{D/P}^{ML}$ Trp variants from the protein complexes, while $NCBD_{D/P}^{L2067W}$ was used to displace the $NCBD_{D/P}^{ML}/CID_{1R}^{ML}$ complex. (e) An example of an ITC binding isotherm where CID_{1R}^{ML} was titrated into $NCBD_{D/P}^{ML}$. All ITC measurements were conducted in the same experimental buffer as above and at 25 °C. Fitting to a one-site two-state binding model yielded the following parameters: K_d = 2.9 ± 0.1 μM and n= 0.8 ± 0.007. (f) The affinities measured by ITC for CID_{1R}^{ML} binding to $NCBD_{D/P}^{ML}$, $NCBD_{D/P}^{T2073W}$ and

$NCBD_{D/P}^{L2067W}$. The error bars denote the standard error from the fit to a two-state function (Supporting Dataset S1f). (g) The fitted parameters for the $NCBD_{D/P}$ Trp variants and $NCBD_{D/P}^{ML}$ from the different experiments.

1. Malatesta, F. (2005) The study of bimolecular reactions under non-pseudo-first order conditions. *Biophysical Chemistry*. **116**, 251–256

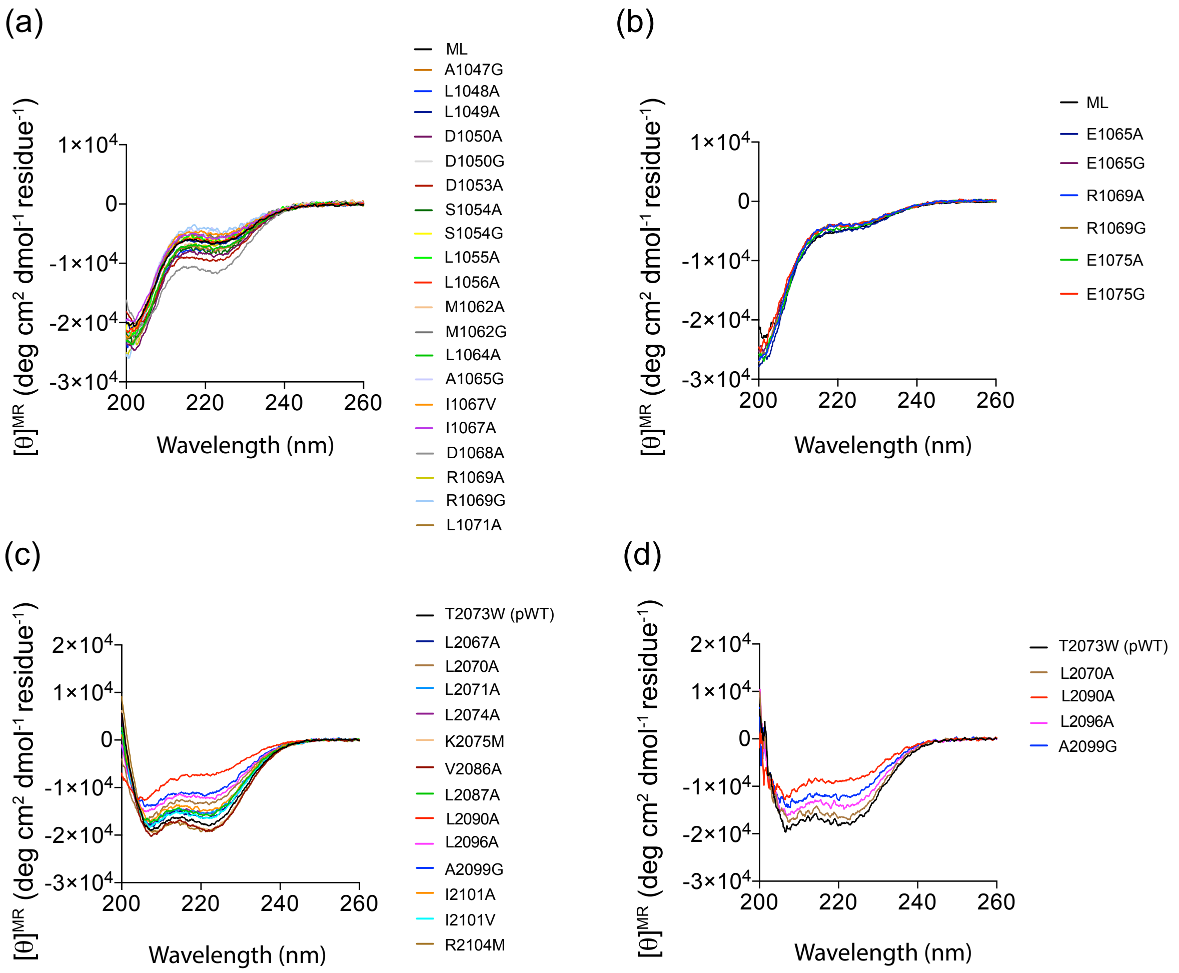

Figure S2. CD spectra of all NCBD and CID variants. All CD spectra were recorded in 20 mM sodium phosphate pH 7.4, 150 mM NaCl at 4°C unless stated otherwise. (a) The CD spectra of all CID$_{1R}$ variants. (b) The CD spectra for all CID$_{Human}$ variants. (c) The CD spectra of all NCBD$_{D/P}^{pWT}$ variants. (d) The NCBD$_{D/P}^{pWT}$ variants L2070A, L2090A, L2096A and A2099G displayed substantially lower helical content as compared to NCBD$_{D/P}^{pWT}$ and the CD spectra of these variants were therefore recorded in buffer supplemented with 0.7 M TMAO. Supplementation with TMAO increased helical content of some variants.

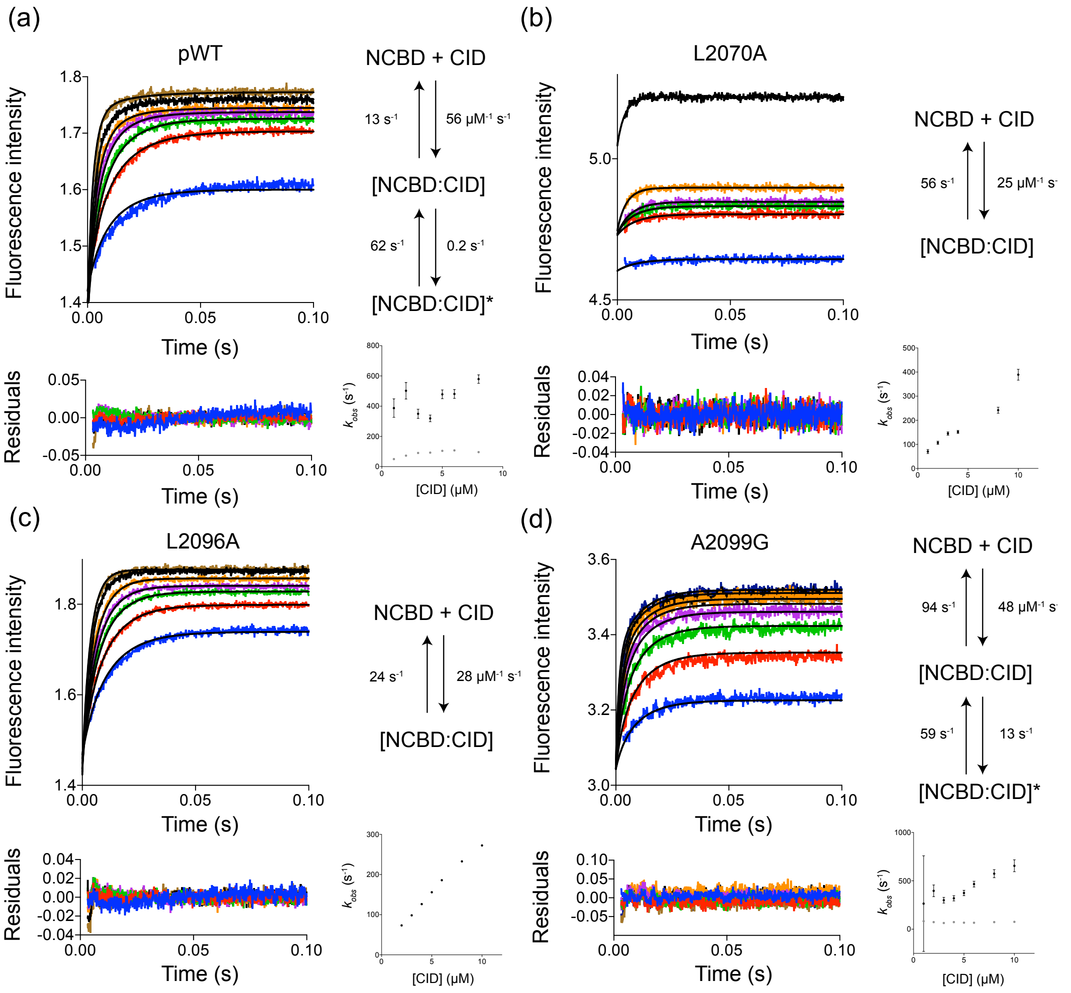

(e)

$NCBD_{D/P}$ variant	CID_{1R} variant	Temperature (°C)	k_{on} (μM^{-1} s^{-1})	K_d (μM) ITC	K_d (μM) Stopped-flow	ϕ-value
pWT	ML	25	-	0.51 ± 0.04	-	-
pWT	ML	20	-	0.25 ± 0.04	-	-
pWT	ML	15	-	0.14 ± 0.03	-	-
pWT	ML	10	-	0.35 ± 0.25	-	-
pWT	ML	4 (extrapolated)	56 ± 6	0.36 ± 0.11	-	-
L2070A	ML	4	25 ± 3	-	2.2 ± 0.2	0.44 ± 0.11
L2096A	ML	4	28 ± 3	-	0.85 ± 0.09	0.8 ± 0.3
A2099G	ML	25	-	5 ± 1	-	-
A2099G	ML	4	48 ± 5	-	-	0.06 ± 0.05

Figure S3. Stopped flow kinetic experiments in presence of 0.7 M TMAO. The stopped-flow binding kinetic and isothermal titration calorimetry experiments were conducted once and in 20 mM sodium phosphate pH 7.4, 150 mM NaCl, 0.7 M TMAO at 4 ºC (Supporting Dataset S1a, e, f). (a) The binding kinetics of $NCBD_{D/P}^{pWT}$ in complex with CID_{1R}^{ML} were biphasic in presence of 0.7 M TMAO and the figure shows the fit to an induced fit model along with the best-fit microscopic rate constants. The graph below the reaction scheme shows the k_{obs} values derived from the fit to a double exponential function plotted against CID concentration. The data shows one linear fast phase (black dots) and one phase with a constant k_{obs} of around 100 s^{-1} (grey dots). The binding kinetics of (b) $NCBD_{D/P}^{L2070A}$ and CID_{1R}^{ML} and (c) $NCBD_{D/P}^{L2096A}$ and CID_{1R}^{ML} was monophasic and was globally fitted to a two-state binding mechanism. The k_{obs} values displayed a linear relationship with CID concentration. (d) The binding kinetics of $NCBD_{D/P}^{A2099G}$ in complex with CID_{1R}^{ML} was biphasic in the

presence of 0.7 M TMAO and was fitted to an induced fit model. The fitted k_{obs} values reveal one faster kinetic phase (black dots) which is linear with CID concentration and one phase which has a constant k_{obs} of around 70 s^{-1} (grey dots). (e) Kinetic and thermodynamic parameters obtained in stopped-flow and isothermal titration calorimetry (ITC) experiments for the protein complexes in (a)-(d). The errors in k_{on} were set to 10 % based on the standard deviation for the $NCBD_{D/P}^{pWT}/CID_{1R}^{ML}$ complex and the errors in k_{off} are standard errors from global fitting of a two-state model for binding. The error of the extrapolated affinity at 4°C for the $NCBD_{D/P}^{pWT}$ was estimated to be 30 %.

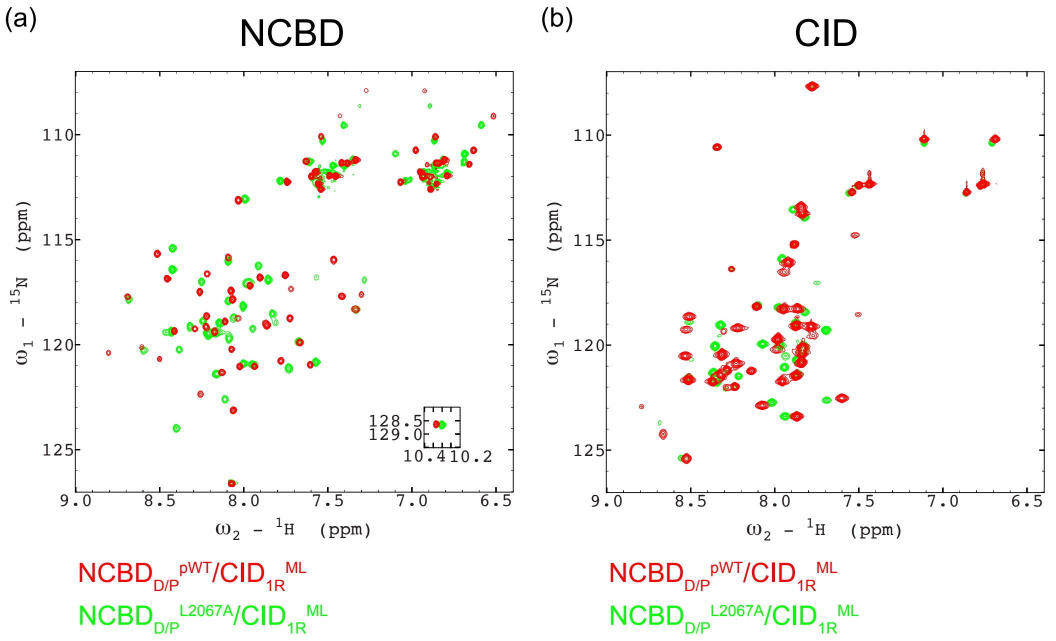

Figure S4. ^{1}H-^{15}N-HSQC spectra for bound CID and NCBD domains. (a) Unlabeled CID_{1R}^{ML} bound to ^{15}N-labeled $NCBD_{D/P}^{pWT}$ (red) and ^{15}N-labeled $NCBD_{D/P}^{L2067A}$ (green). The inset shows the tryptophan side chain peak. (b) ^{15}N-labelled 1R CID_{1R}^{ML} bound to unlabeled $NCBD_{D/P}^{pWT}$ (red) and $NCBD_{D/P}^{L2067A}$ (green).

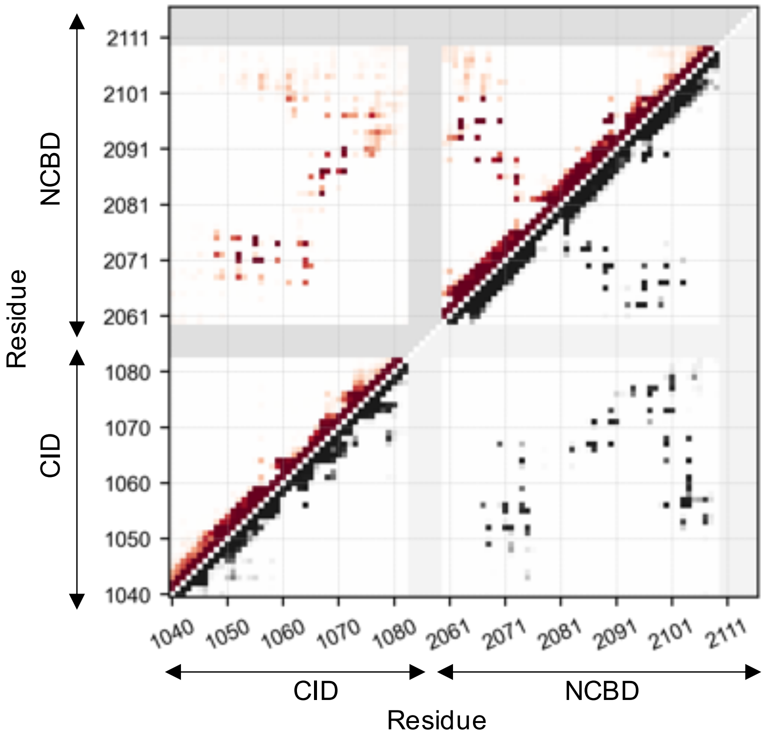

Figure S5. Map representing the contact probability between each pairs of residues in the ancestral native state (lower right, gray) and in the ancestral TS (upper left, red) ensembles. Probability goes from 0 (white) to 1 (dark gray/red); regions, involving residues which are present in the human but not the ancestral complex, are shaded with gray to be consistent with Fig. 3 in the main text.

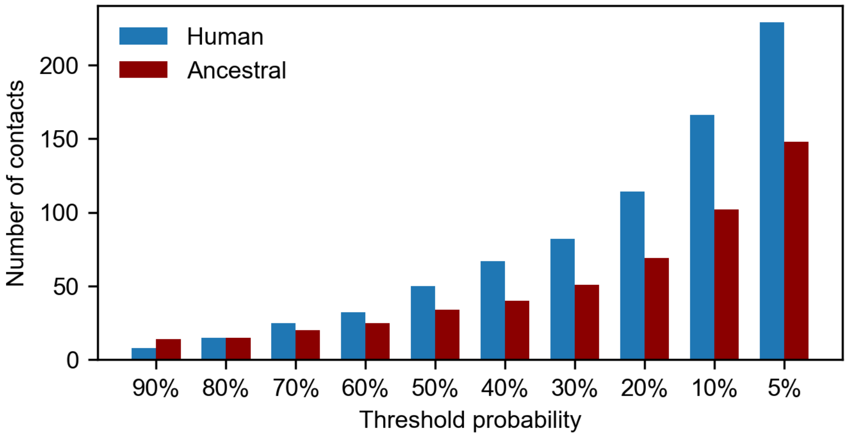

Figure S6. Number of intermolecular residue-residue contacts present in the human/ancestral TS ensemble with a probability higher than a threshold probability.

Supporting Video S1. Representative structure of the Cambrian-like TS. The residues associated to the ϕ-values used as restrains in the MD simulations are highlighted as sticks and colored according to the measured ϕ-value, from red to yellow to blue.

Supporting Dataset S1. A file containing all numerical parameters derived from the different experiments performed, along with fitted and propagated errors.
